# TP53 in Biology and Treatment of Osteosarcoma

**DOI:** 10.3390/cancers13174284

**Published:** 2021-08-25

**Authors:** Kamil Jozef Synoradzki, Ewa Bartnik, Anna M. Czarnecka, Michał Fiedorowicz, Wiktoria Firlej, Anna Brodziak, Agnieszka Stasinska, Piotr Rutkowski, Paweł Grieb

**Affiliations:** 1 Small Animal Magnetic Resonance Imaging Laboratory, Mossakowski Medical Research Institute, Polish Academy of Sciences, 02-106 Warsaw, Poland; ksynoradzki@imdik.pan.pl; 2 Department of Experimental Pharmacology, Mossakowski Medical Research Institute, Polish Academy of Sciences, 02-106 Warsaw, Poland; amczarnecka@imdik.pan.pl (A.M.C.); astasinska@imdik.pan.pl (A.S.); pgrieb@imdik.pan.pl (P.G.); 3 Institute of Genetics and Biotechnology, Faculty of Biology, University of Warsaw, 02-106 Warsaw, Poland; ebartnik@igib.uw.edu.pl; 4 Institute of Biochemistry and Biophysics, Polish Academy of Sciences, 02-106 Warsaw, Poland; 5 Department of Soft Tissue, Bone Sarcoma and Melanoma, Maria Sklodowska-Curie National Research Institute of Oncology, 02-781 Warsaw, Poland; s071395@student.wum.edu.pl (W.F.); piotr.rutkowski@coi.pl (P.R.); 6 Faculty of Medicine, Medical University of Warsaw, 02-091 Warsaw, Poland; 7 Laboratory of Centre for Preclinical Research, Department of Experimental and Clinical Physiology, Medical University of Warsaw, 02-097 Warsaw, Poland; anna.brodziak@wum.edu.pl; 8 Department of Oncology and Radiotherapy, Maria Sklodowska-Curie National Research Institute of Oncology, 02-781 Warsaw, Poland

**Keywords:** TP53, osteosarcoma, gene therapy, animal models, pharmacological modulation

## Abstract

**Simple Summary:**

Treatment of osteosarcoma, apart from chemotherapy modifications, has not changed for approximately 30 years. Similarly, as in other tumors, mutations in the TP53 gene are often observed in osteosarcoma. In this article, we highlight the possibility of targeting p53 in the treatment of osteosarcoma. We collected data on mutations in this gene founded in patients-derived samples. We describe animals with TP53 dysfunction, which may constitute preclinical models. We put emphasis on several molecules which act on p53 protein or its activity. We also highlight gene therapy approaches. Although many of the therapies are at an early stage, they offer hope for a change in the approach to osteosarcoma treatment based on *TP53* targeting in the future.

**Abstract:**

The *TP53* gene is mutated in 50% of human tumors. Oncogenic functions of mutant *TP53* maintain tumor cell proliferation and tumor growth also in osteosarcomas. We collected data on *TP53* mutations in patients to indicate which are more common and describe their role in in vitro and animal models. We also describe animal models with *TP53* dysfunction, which provide a good platform for testing the potential therapeutic approaches. Finally, we have indicated a whole range of pharmacological compounds that modulate the action of p53, stabilize its mutated versions or lead to its degradation, cause silencing or, on the contrary, induce the expression of its functional version in genetic therapy. Although many of the described therapies are at the preclinical testing stage, they offer hope for a change in the approach to osteosarcoma treatment based on *TP53* targeting in the future.

## 1. Introduction

Osteosarcoma (OS) is a primary malignant tumor of the skeleton, characterized by the formation of immature bone or osteoid tissue by the sarcoma cells that develop mostly in the long bones. Localized (non-metastatic) OS, if treated in specialized sarcoma centers with surgery along with pre- and postoperative chemotherapy, is cured in 60–70% [1]. Still, survival rates are as low as 15% for metastatic patients because multidrug resistance to chemotherapy is a frequent problem in this disease [2]. Complete surgical removal of all metastatic (pulmonary) nodules remains the only curative treatment option. Oligometastatic and oligoprogressive disease is being treated with radiotherapy [3,4]. Despite numerous modifications of chemotherapy regimens for metastatic OS cases, outcomes are still unsatisfactory in this neoplasm. No successful targeted therapies have been introduced in routine clinical practice for OS so far [4,5]. In OS, genomic alterations and signatures associated with sensitivity to treatment with DNA damage response (DDR) inhibitors are frequent, including those similar BRCA1-related and alternative lengthening of telomeres (ALT) pathway-related. As a result, PARP inhibitor (Olaparib), in combination with ATR inhibitor (ceralasertib), has been evaluated in a trial (NCT04417062). Other tested molecules include sorafenib, everolimus [6], regorafenib evaluated in the REGOBONE study, apatinib, and camrelizumab [7]. An important role of immune-mediated therapies is thus expected in OS [8,9].

The oncogenic functions of mutant *TP53* are very similar in sarcoma and multiple cancers. In the sarcoma field xenograft models expressing mutant *TP53* in pleomorphic rhabdomyosarcomas, the tumor profile was even more aggressive and with greater metastatic potential than p53 null or p53 wild-type tumors. In particular, p53G245C and p53R273H mutations were reported to promote a more aggressive phenotype with an enhanced malignant potential, high cell viability, and proliferation, enhanced cell migration [10,11,12]. All these molecular data suggest that *TP53* is a promising therapeutic target in OS. Selected therapies aimed at p53 reactivation are expected to suppress OS [13]. The development of clinical trials including small molecules that could restore the wild-type conformation of p53 and gene therapy combined with chemotherapy, radiation therapy, or conventional surgery are expected in the future. Despite great progress in gene therapy, it is still most commonly in the experimental stage, not in the actual clinical application except for Gendicine, approved in China [14,15].

This article aims to review the current knowledge of the role of *TP53* in OS and its potential as a therapeutic target. We review different types of experimental therapeutic approaches, including gene therapy and pharmacological modulation of p53. We also review the OS animal models particularly useful for studying *TP53*-targeted treatments.

## 2. Structure and Functions of TP53

The *TP53* gene spreads over 13 exons mapped to region 17p13.1 [16]. *TP53* encodes at least 12 different isoforms [16]. Isoforms may regulate various processes by enhancing and inhibiting wild-type p53 transcriptional and growth-suppressing activities [17,18]. In normal conditions, the activity of the ubiquitin ligase MDM2 inhibits p53 transcriptional activity and promotes its degradation. Additionally, p53 activity is modulated through posttranslational modifications [19,20]. In its active state, p53 is a homotetramer and modifies gene expression by binding to its specific target sites, interacting with other proteins and DNA. Each monomer consists of two acidic N-terminal transactivation domains [21], a proline-rich domain, a core DNA-binding domain, an oligomerization domain, and a C-terminal regulatory domain containing nuclear localization signals that mediate the migration of the protein into the cell nucleus [21,22,23,24].

**Figure 1:**
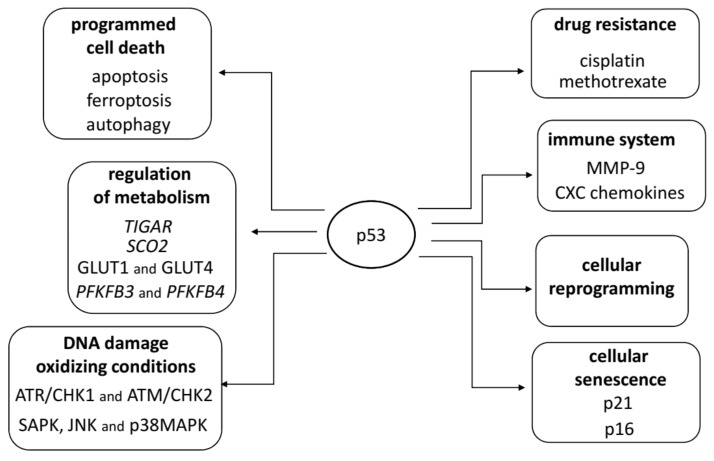
The main functions of p53 in the cell life and death.

The p53 protein is a central hub for activation of numerous stress-induced pathways, including DNA damage, senescence, metabolism; it is also engaged in cellular death, cellular reprogramming, and drug resistance. Below, we outline these areas of the p53 activity in the cell (Figure 1).

Activation of p53 is stimulated by DNA damage and is dependent on ATR/CHK1 and ATM/CHK2 signaling. Oxidizing conditions could also stimulate p53 activation through stress-activated kinases (SAPK, e.g., JNK and p38MAPK) independently from the DNA damage response [25,26].

The p53 protein is engaged in programmed cell death. It activates several genes encoding apoptotic proteins (PIG, BAX, Fas/Apo1, Killer/DR5, PUMA, or Noxa) [27,28,29,30,31,32,33,34,35]. Apoptosis resulting from induction of the caspase-3 cascade and increased Bax/Bcl-2 ratio was observed in OS cell line U2OS after treating with cisplatin [36]. In autophagy, p53 plays an ambiguous role depending on its localization. P53 activates several genes in the nucleus encoding pro-autophagic factors, e.g., TSC2, PTEN, AMPK, DRAM (damage-regulated autophagy modulator) [37]. Cytoplasm-localized p53 inhibits autophagy by down-regulation of AMPK and up-regulation of mTOR activity [38,39]. In another programmed cell death process called ferroptosis, p53 regulates cell sensitivity of ROS-induced ferroptosis by regulating transcription of *SLC7A11* membrane transporter, the activity of GPX4, and in consequence, accumulation of ROS [40].

p53 regulates transcription of cellular senescence marker p21, which mediates p53-induced cycle arrest and promotes senescence, including nucleotide-associated senescence [41,42,43,44,45,46,47]. Overexpression of p21 protein in OS cell line (U2OS) sensitizes these cells to apoptosis caused by cisplatin [36]. Another senescence protein marker, p16 was considered to be a predictor of the response to neoadjuvant chemotherapy in a Caucasian population of patients with high-grade OS [48]. p53 can maintain cell growth or senescence response after telomere dysfunction; its activity depends on the p16 protein level [49].

p53 plays an important role in inducing pluripotency. Silencing of p53 resulted in increased reprogramming efficiency in murine and human cells [50,51]. The absence of p53 activity leads to the high-efficiency formation of cancer-derived induced pluripotent stem cells (iPSCs), which is reversed in cells with active *wt* p53 [52]. The activity of the mutated version of p53 (R172H) or depleted expression of *wt* p53 also reduces the number of transcription factors necessary to generate iPSCs from embryonic fibroblasts [50,53].

p53 is a regulator of various metabolic pathways. It reduces glucose uptake, represses transcription of *GLUT1* and *GLUT4* [54]. The binding of p53 in two activation sites of the *TIGAR* gene was confirmed in osteosarcoma Saos-2 and U2OS cell lines [55]. Elevated expression of Tigar results in inhibition of glycolysis by decreasing the intercellular fructose-2,6-bisphosphate level [56]. p53 represses the expression of *PFKFB3* and *PFKFB4* genes, leading to inhibition of glycolysis and an increase of ribonucleotide generation via the pentose phosphate pathway [56,57]. p53 regulates mitochondrial aerobic respiration. It enhances mitochondrial oxidative phosphorylation via the expression of the *SCO2* (Synthesis of Cytochrome c Oxidase 2) gene [58].

In p53-deficient tumors, restoration of p53 activity results in an inflammatory reaction involving neutrophils, macrophages, and NK cells [59]. Altered p53, such as in R175H, R273H, and D281G, up-regulates the expression of chemokines CXCL5, CXCL8, and CXCL12 [60]. Epigenetic down-regulation of CXCL12 in OS cells results in acquiring the ability to metastasize. In patient-derived samples, a low level of CXCL12 correlates with a low level of lymphocytes infiltrating the tumor and poor overall survival of patients with OS [61]. Mutated p53 may remodel the tumor microenvironment. Several mutant p53 forms increase expression of MMP9, the enzyme that degrades extracellular matrix ECM [62]. Meta-analysis studies show that metastasis occurs more frequently in patients with MMP-9 overexpression, and the prognosis is worse than in OS patients with low MMP-9 expression [63].

Altered p53 proteins may modify tumor-transformed cell response to treatment. U2OS cells with active *wt* p53 were more sensitive to cisplatin than A431 cells (bearing p53 R273H) [64]. Radiological and histological evaluation of tumors from OS patients reveals that loss of heterozygosity of the *TP53* gene is associated with resistance to preoperative chemotherapy (cisplatin, methotrexate) [65]. Chemosensitivity of OS cells to cisplatin is enhanced by p14^ARF^ [66]. This protein can bind to the MDM2, thus inhibiting the nuclear export and p53-ubiquitination and increasing the stability of the p53 [67,68].

## 3. Malfunction of TP53 in Osteosarcoma

The *TP53* gene is mutated (somatic mutations) in over 50% of human neoplasms, the mutations occur mainly in the DNA-binding domain, but over 20% are outside it [69]. Most of the mutations are missense, but about 10% are nonsense mutations, which lead to the production of truncated p53 proteins [70]. In 60% of neoplasms with missense *TP53* mutations, the second *TP53* allele is deleted [71].

**Figure 2:**
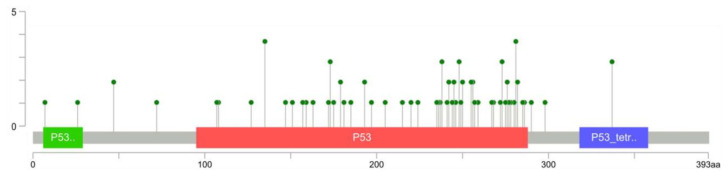
The primary structure of p53 protein with marked domains and amino acid codons showing the number of missense mutations. The X-axis represents the p53 amino acid sequence, Y-axis number of found substitutions. Transactivation domain (green); Core domain which can bind DNA (red); oligomerization domain (blue). Figure created basing on the data published in references [80,81,82,83,84,85,86,87,88,89,90,91,92,93] using cBioPortal Mutation Mapper [94,95].

Mutations in the *TP53* gene may exert three possible effects. First, if transformation occurs in two alleles or when it appears in one allele but the second one is lost, it depletes the ‘guardian’ p53 function. Secondly, if mutated p53 controls its co-expressed wild-type form preventing its binding to DNA, such a situation is called the dominant-negative effect. Thirdly, mutated p53 acquires a new activity absent in the native form of protein, i.e., gain-of-function [72]. It should be noted that these effects are not necessarily exclusive, e.g., R175H mutation may result in a gain-of-function [73] and as well in a dominant-negative effect [74]. Multiple *TP53* gene mutations were found in the studies on OS patients (Figure 2, Table S1). Most commonly substitutions in four amino acids were noticed for codons 135 and 281; three for 173, 238, 248, 250, 273, 337; two for 47, 179, 193, 242, 255, 256, 279, 282. Several mentioned alterations play a crucial role in the proper action of p53—amino acids in positions 248, 273, 282 contact DNA. Residues in positions 175, 220, 245, 249 stabilize the protein structure [75,76,77].

Mutations in codons 175, 220, 245, 248, 273, and 282 are termed ‘hotspots’ and were described as the most frequently altered (in general) positions in P53 protein, based on datasets The Cancer Genome Atlas (TCGA), International Cancer Genome Consortium (ICGC) and International Agency for Research on Cancer (IARC). The most frequent amino acid substitutions in these codons are classified as non-functional [78,79]. Mutations in hotspot codons are well characterized compared to rare variants such as I255T or A159N. The most common mutations found in OS patients or cell lines are described further in the text.

### 3.1. Osteosarcoma in Li-Fraumeni Syndrome

Li–Fraumeni syndrome (LFS) is a rare, autosomal dominant hereditary disorder—where mutations in the *TP53* gene occur in about 70% of the cases [96,97]. Germline mutations in the *TP53* gene lead to Li-Fraumeni syndrome, in which different tumors appear in people of different ages—very early (infants or young children) in endoderm and mesoderm-derived cells (this includes OS) but much later, in old age, in endoderm derived cells [77,98,99]. Several clinical classification schemes have been established, such as classic Li–Fraumeni syndrome (LFS) [100], Li–Fraumeni-like syndrome (LFL) [99], and criteria developed by Chompret [101].

In a study including 525 families diagnosed with Li-Fraumeni (at least a three-generation pedigree in the family history), according to various criteria classification, mutations in the *TP53* gene occur in 14% to 56% of cases. The most common types of cancer found were sarcoma (32.7%), brain tumor—including choroid plexus (9.3%), breast cancer (31.2%), or adrenocortical carcinoma (9%), bone tumors were rare and found in 0.7% of the studied group. In another large study, including 474 families, germline alteration of *TP53* was found in 82 families (17%) [102]. Most mutations are missense type (67%) and lead to loss-of-function of the *TP53* gene. Deletions removing the entire *TP53* locus, the promoter, and exons 1–10 were also observed [102].

NGS sequencing of tumor samples revealed 32 variants of the *TP53* gene in a group of 765 patients. Among missense mutations, the most frequent alteration was the common exonic variant P72R. Other more frequently observed mutations were P47S, Y107H, R273H, R248Q [82]. P47S is a variant frequently present in people of African descent. This mutation reduced cell death and impaired apoptosis induced by cisplatin in vitro, compared to cells with expression of *wt* p53 [103]. Mutations R175H, R248Q and R273H are gain-of-function [104]. The mutant p53 proteins (R175H, R248W, and R273W) were checked in cells that lack wild-type p53 (murine fibroblast 10(3) and human osteosarcoma Saos-2). Their activity results in growth efficiency in agar or enhancing tumorigenic potential in nude mice, confirming their gain-of-function type [105]. These three mutations (R175H, R248W, and R273W) exert a dominant-negative effect. R175H and R273H increased resistance of tumor cells (H1299 cell line) to low cisplatin concentration (2.5 µg/mL) [106]. The activity of p53 R175H decreases doxorubicin-induced apoptosis response in the Saos-2 cell line [107]. In the same cell line, the activity of p53 R273H was found to induce resistance to drug-induced apoptosis (methotrexate and doxorubicin) through down-regulation of procaspase-3 [108]. Expression of gain-of-function *TP53* R172H (corresponds to human R175H) in mouse p53 null fibroblasts or heterozygous OS cell lines results in overexpression of Pla2g16 protein. Increased level of Pla2g16 leads to a more aggressive and metastatic cell phenotype [109,110]. Heterozygous mice with high expression mutated p53 (R172H) develop metastatic OS. Metastasis was observed in lymph nodes, lungs, liver, and brain [111]. In a mouse model where osteoblast cells express p53 R172H, but the tumor microenvironment cells remained *wt* p53, lung metastasis was still observed [112].

Mutations in the *TP53* gene found in LFS patients by Birch et al. were localized in codons E180K, R175H, Y220C, D245G, and R248Q [99]. The Y220C mutation is localized outside the DNA-binding surface [113]. In yeast assays, it was categorized as a loss-of-function type [114]. This mutation causes a structural change in the protein and the formation of a hydrophilic cleft [113]. In silico analysis and virtual screening allowed designing a carbazole derivative (PhiKan083). PhiKan083 stabilizes the structure of the mutated protein and prolongs its half-life [115]. Mutation G245D (gain-of-function) promotes invasive cell growth of the UM-SCC-1 cell line (head and neck squamous cell carcinoma), and it was assessed by in vitro and in vivo experiments. This mutation induces an aggressive growth phenotype, up-regulation of *FOXM1* expression, and inhibiting AMPK and FOXO3a activation [116]. Tumor cells expressing p53 G245D induced large pulmonary metastases in nude mice [117]. Molecular dynamics simulation reveals that this mutation changes p53 conformation by introducing a novel zinc-binding site [118].

### 3.2. TP53 Alterations in Osteosarcoma Patient-Derived Samples

Initially, *TP53* mutations were only detected in 20% of OSs, but currently, this frequency is believed to be much higher—from 47–90%. Moreover, numerous structural alterations involve this gene, especially the translocation of the first intron [119]. Patients with *TP53* mutations had poorer overall survival. Lorenz et al. [93] found that rearrangements were the major mechanism of p53 inactivation, but mutations and *MDM2* amplifications were also observed. Somatic *TP53* mutations are an unfavorable prognostic marker for 2-year survival in OS patients [120].

Chen et al. [89] performed whole-genome sequencing of 20 OS samples with matched normal tissue controls and found that the p53 pathway was affected in all of them; in nine samples, translocations in the first intron of the *TP53* gene were detected. In one case, an *MDM2* amplification was found, and as MDM2 is a negative regulator of the p53 protein, this would lead to down-regulation of the protein. They extended their sequencing analysis to 32 further OS samples and found that 50% had rearrangements of the *TP53* gene, 38% had missense (H179Q, Y205C, R337H, R248Q, R273H, E285K) or nonsense mutations (codons 169 and 211), 6% a *TP53* gene deletion and again 3% (1 case) had an *MDM2* gene amplification. Two fusions of the *TP53* gene with *ASIC2* or *SFSWAP* genes were also observed. The rearrangements involving intron 1 of *TP53* have not been found in other neoplasms, thus appearing specific for OS [69]. Mutation H179Q causes a dominant-negative phenotype. Cells with the expression of this mutant protein were immortal and showed attenuation of G1 checkpoint function and loss of expression of p21 protein [121]. Expression of p53 Y205C in cell line H1299 (lung cancer-metastatic derived) results in the loss of the protein’s transactivation ability [122]. Amifostine can restore p53 Y205C function in a yeast-based assay [123]. Some studies report that the E285K mutation is a loss-of-function type [114,124]. Arginine in the 337 position is localized in the tetramerization domain, and its substitution results in a functionally defective protein [125]. R337H is common in south-eastern Brasil [126,127] and is associated with the occurrence of adrenocortical cancer, choroid plexus, and OS tumors in young patients [128,129]. This mutation also has a high incidence in breast cancer patients in Brasil [127].

Mirabello et al. [130] analyzed the frequency of germline mutations of 238 genes in 1244 OS patients. For 765 cases, all *TP53* exons were sequenced; mutations were found in 9.5% of young patients but none in patients aged 30 years or older. A more extensive paper by this group confirmed these findings, though a *TP53* germline mutation was also found in one 39-year-old patient. Frequent mutations were: P47S, Y107H, R273H, R248Q, and P36, R213 in the exonic splice site [82]. The first two (R273H, R248Q) were classified as non-functional by the IARC database and Y107H as partially functional.

Rare mutations were reported by Bousquet et al. [85], and seven tumors from young patients with OS were found. In three of them (patients aged 12, 15, and 16 years), the *TP53* gene mutations were deletions, missense type (A159N), STOP codon (192), and splice site mutation (307). The authors maintain these alterations probably lead to degradation of changed *TP53* transcript or result in expression of a truncated form of the *TP53* gene.

WGS data from 25 OS tumors collected from patients of both sexes in the age range between 8–79 years revealed mainly rearrangements in intron 1 and intron 9. The final transcripts lose essential parts of the protein, so these aberrations result in loss-of-function. Identified point somatic missense mutations were C238F, I255T, R273L, C275Y, D281E. Based on yeast assays or structure predictions, these mutations were classified as loss of protein function. The loss-of-function type was confirmed by Jordan et al. [114] for mutations C238F, D281E, and R273L, R273L, while C275Y is a gain-of-function mutation [104], mutation C238F localizes in the L3 loop [93,114]. Intriguingly, the expression of C238F may also result in a gain-of-function. Its expression in head and neck squamous cell carcinoma cells shows up-regulation of FOXM1, high invasive potential in vitro, and gross pulmonary metastases after injection into nude mice [116]. D281E mutation based on luciferase and yeast plate color assay—was classified as non-functional [114]. It was also discovered in an Iranian 43-year-old male patient who had OS and LFS [131].

Saba et al. [132] analyzed 148 OSs (both pediatric and adult patients) by in-depth sequencing. They found that rearrangements in the *TP53* intron 1 led to the fusion of the promoter to various genes, and showed by transcriptomic analyses that this led to the activation of numerous genes, also ones belonging to the tumor protein p53 pathway, and the rearrangement leads to reactivation of many of these genes. The interesting findings of that paper are that this rearrangement is an early event in OS genesis, and it is present in all OS cells, thus could be a potential target for future therapies.

Two mutations in the *TP53* gene—P72R and R248W were found in human osteosarcoma cancer stem cell line 3AB-OS. The authors classified these alterations as gain-of-function. Cells bearing these mutations proliferate rapidly [133]. In other cell lines (IOR/OS15, MG-63, KPD, and Saos-1) derived from the human OS, several fusion transcripts with *TP53* were found. These transcripts consisted of the first exon of *TP53* joined with exons of *VAV1*, *DDX39B, SAT2, EMR1*, *PPRAD* genes. A series of truncated transcripts of *TP53* were also observed. In the IOR/SARG cell line, the stop codon at position 205 results in the loss of a part of the DNA—binding and C-terminal domains [93].

## 4. Animal Models

The development of pre-clinical systems is crucial to enable basic research on the role of p53 in OS and provide a platform for testing the potential therapeutic approaches. Several animal models mimicking dysregulation of p53 were already proposed [134]. We list and summarize them in Table 1 and broadly describe them below. Numerous mouse models were developed to study the effects of missense mutations on p53 activities in vivo. The *Trp53* (mouse homolog of human *TP53*) knockout (also known as p53 KO) mutant mice develop tumors at three to six months of age [135]. They are thought to be suitable for use in applications related to the study of familial breast cancers such as Li-Fraumeni syndrome and research of lung, brain, and bone tumors, lymphoma, and leukemia. However, their usefulness for sarcoma research is limited due to the spectrum and frequency of different tumor types developed in these mice. Other p53-deficient mouse models have been developed to study sarcomagenesis. In particular, mice develop spindle cell sarcomas and pleomorphic sarcomas if *Trp53* is inactivated by Cre-loxP-mediated recombination. Additionally, mesenchymal sarcoma stem cells (Sca-1low) have been isolated from these animals, but these mice are deficient in p53 and pRb, making detailed analyses difficult [136].

It was postulated that the human Li-Fraumeni syndrome characterized by heterozygous *TP53* mutations would be modeled more accurately rather by heterozygous than homozygous animal models [134]. Indeed, heterozygous mutant *Trp53* mouse models, which require LOH before tumorigenesis occurs, develop a spectrum of tumors that is more similar to humans. While homozygotes develop lymphomas, the most frequent tumors in heterozygotes were OSs and soft tissue sarcomas [135]. In this approach, tumors develop later than in homozygous animals (half of the homozygotes develop tumors by 45 months of age and heterozygotes by 12 months), which results in higher costs and longer experiment duration [135].

The role of *Trp53* missense mutations was investigated in transgenic mice with knock-in alleles that mimic the hot spot mutations found in human *TP53*. p53 R172H and p53 R270H mutations were introduced to the mouse p53 corresponding to amino acids 175 and 273 in human p53. These are most often mutated in human carcinomas [137]. The spectrum of tumors that develop in mice depends on the various mutant alleles in the model. For example, the ‘DNA contact’ mutant p. R270H (p. R273H in humans) results in a high frequency of carcinomas, whereas the ‘structural’ mutant p. R172H (p. R175H in humans) predominantly results in OSs. In contrast, p. R175P are defective in activating genes associated with apoptosis without impairing growth arrest.

Compared with *Trp53−/−* mice, these knock-in models display a higher degree of heterogeneity in the spectrum of tumor types with more frequent carcinomas (than sarcomas). On the other hand, homozygous *Trp53−/−* mice are highly prone to develop T-cell lymphomas and selected sarcomas [138]. Although the loss of *Trp53* in mice results in thymic lymphoma as the dominant tumor type, in humans, these lymphomas are less frequently *TP53* mutated/dependent [139].

Several p53-deficient animal models were also developed in rats. One example is the *Tp53*-deficient Dark Agouti rat [140]. In this model, homozygotes develop angiosarcomas and lymphomas, and their lifespan is limited to 6 months. Heterozygotes live longer (up to 12 months) and develop a broader spectrum of tumors; however, angiosarcomas and lymphomas are also the most common, and OSs are rare [141].

Another rat model, obtained in the Fischer-344 (F344) rat (F344-Tp53^tm1(EGFP-Pac)Qly^/Rrrc (F344-Tp53) strain) develops predominantly meningeal sarcomas and OSs [142]. In homozygotes, the frequency of OSs was 57%, and in heterozygotes—36%. The spectrum of tumors was also broader in heterozygotes than in homozygotes. Moreover, OS pulmonary metastases were also found in this model.

Tp53C273X/C273X Wistar rats carry a single point mutation that introduces a premature stop codon. Homozygotes develop mostly angiosarcomas, while heterozygotes develop OSs and less frequently angiosarcomas and B-cell lymphomas [139]. Metabolically active tumors are present in various locations, including the brain, head and neck, abdomen, and extremities [143,144]. Another Tp53 knockout rat generated on the Sprague Dawley background also develops mostly sarcomas. However, the spectrum of tumors seems to be generally broader than in the Wistar knockout rats. OS is the most frequently observed type of tumor in homozygotes, but still, it constitutes only 18% of all tumors [145].

In general, the rat models seem to be a promising tool for studying the role of p53 malfunction of OS. Some of these models predominantly develop OSs in a relatively short time. The rat is also easier to handle and provides better perspectives for advanced imaging methods that would allow precise tracking of potential therapeutic approaches.

Another recently proposed model was developed in zebrafish (*Danio rerio*). In one of them, mutation p53I166T was induced by exposure of the embryos to gamma irradiation [146]. Both heterozygous and mutant fish developed soft tissue sarcomas with high penetrance but most frequently spindle cell tumors. Recently, a zebrafish *Tp53* knockout was also generated [147]. These knockouts develop a wide range of tumors, including angiosarcomas, malignant peripheral nerve sheath tumors, germ cell tumors, or leukemias. Despite the limitations of the zebrafish models, they enable large-scale high throughput testing of potential therapeutics and direct visualization of tumor growth. Such a model could be valuable for the initial evaluation of the potential drugs.

On the other hand, experiments or trials performed on spontaneously developing tumors in pet dogs could be the final step of translating a potential therapeutic approach into human clinical trials and then routine applications [148]. OSs occur at least ten times more often in dogs than in humans (with an incidence rate estimated to be around 14 cases/100,000 dogs according to [149]). A review by Regan et al. [148] reported that OS was the second disease with the largest number of active clinical trials in pet animals. Importantly, *TP53* mutations were reported in canine OSs, e.g., Kirpensteijn et al. [150] *TP53* mutations were found in 40% of OSs.

## 5. p53 as a Therapeutic Target in Osteosarcoma

Though many papers are devoted to this subject, the p53 protein is not considered an easy target for cancer therapy. The problems are caused by the numerous different mutations, their varied effects on the protein, and the gain-of-function that some of them convey, and also even if the wild-type protein is present (if the wild-type *TP53* gene is not lost), the mutated protein will affect the structure of the whole tetramer, which will not function properly [77].

Of course, different therapies would need to be applied if the p53 protein is missing and mutated and has acquired new activities [77]. Although *TP53* is altered in many or most sarcomas, it is not an obvious nor easy target. In some reviews of potential new therapies for sarcoma, it is not even mentioned. As little has happened in the last 30 years, new therapies are needed, and survival rates for patients have essentially remained the same [153].

The way to treat OS by acting on p53 could be indirect by acting on its interactors. An example is shown by Samsa et al. [154]. *JAB1* (JUN activation domain-binding protein) is overexpressed in OSs, and its knockdown decreases the oncogenic properties of OS cell lines. Moreover, a small molecule inhibitor of the protein affects the viability of OS cells, and Samsa et al. have developed an animal model which shows that JAB1 oncogenesis depends on *TP53*. It is suspected that targeting JAB1-associated miRNAs can be used for clinical intervention in OS.

Another OS treatment method may be miRNA-based therapeutics. miR-34 family members (miR-34a, miR-34b, miR-34c) are established regulators of tumor suppression, and their expression is transcriptionally controlled by p53 [155,156,157,158]. It was reported that miR-34a expression in OS is significantly down-regulated, promoting oncogenic properties of OS cells [159,160,161,162]. miRNA-34a is reported to increase cisplatin sensitivity in OS cells in vitro and be a key regulator in the dedifferentiation of OS [161,163]. Phase I clinical trials have investigated liposome-based delivery of the down-regulated miR-34a for advanced stages of multiple solid and hematological malignancies. However, due to reported immune-related adverse effects, the study had to be terminated [158,164].

Ganjavi et al. [165] used adenoviral vectors to introduce the *wt* p53 protein-coding gene into four sarcoma lines. They found that the cell lines became sensitive to cisplatin and doxorubicin, though these were not OS lines. The crucial problem in OSs and therapy aimed at *TP53* is that in many OSs, the rearrangements lead to the activation of numerous genes. This is to some extent due to rearrangements involving the *TP53* gene promoter, it is not clear whether attempts to reactivate or introduce a normal copy of the gene would be successful [132]. Numerous papers deal with targeting mutated *TP53* and/or its interactors; however, they rarely refer to OS.

For tumors with decreased p53 levels, therapy could act on its interactors, which regulate p53 activity—MDM2 can affect p53 localization and promote its degradation; moreover, it can block its ability to transactivate by binding to the N-terminal domain of the p53 protein [13]. MDMX also binds to the N-terminal transactivating domain of p53. Various inhibitors of MDM and MDMX have been proposed, but as many OSs do not contain an active p53 molecule addressing its inhibitors may not be the solution in most cases. In neoplasms with mutant p53 small molecules are used to ‘normalize’ the protein; however, this is complicated as there are numerous *TP53* mutations, which may not be relevant for osteosarcoma [166].

The literature on potential treatments of various types of cancer through acting on the p53 protein is abundant. It is essentially based either on stabilizing the wild-type form by acting on its destabilizers—mainly MDM2 (or on acting on the mutant p53 protein to restore its normal function) [167]. The former method may not apply to most OSs, as MDM2 up-regulation is relatively rare. As for the latter—it could apply to the tumors in which p53 is mutated, though not to the ones where the protein is absent due to rearrangements of the *TP53* gene. Moreover, as *TP53* mutations are rarely recurrent, and there are many different ones, this restoration would have to be tested for numerous mutations to see how effective it would be.

One of the molecules which could restore wild-type activity is PRIMA-1 (2,2-bis(hydroxymethyl)1-azabicyclo(2,2,2)3-octan-3-one) (PRIMA is an abbreviation for p53 reactivation and induction of massive apoptosis) and its methylated form PRIMA-1MET [167]. Both compounds inhibit the growth of various mutant p53 expressing cell lines in vitro and have shown low toxicity in animal models. Interestingly, PRIMA-1 has shown synergistic effects with camptothecin in an OS cell line [167].

Moreover, two compounds with potential activity to reactivate mutant p53, APR-246, a methylated structural analog of PRIMA-1, and COTI-2, a third-generation thiosemicarbazone, have been shown to work in pre-clinical models [168]. These two compounds have been used in phase I clinical trials but not for OSs [168]. Bykov et al. [70] list 20 different p53 targeting compounds, divided into several groups, but all are listed as experimental/and or pre-clinical. Moreover, the authors comment on the observed non-p53 related activities of many of these compounds, noting that this is expected of small molecules. The problem with *TP53* mutations is that there are so many different ones. Still, some attempts to target specific mutations have been described, including a carbazole derivative molecule PhiKan083 which stabilizes the eighth most common mutation Y220C [70].

Different strategies are required to obtain stable p53 for the relatively rare nonsense mutations; this would require drugs, such as aminoglycosides, which promote translational readthrough, which would lead to a normal protein. Another approach would be to target NMD (nonsense-mediated decay) of nonsense-codon-containing mRNAs. At least one such inhibitor, NMD114, has been shown to improve the stability of the mRNA for p53 [70].

There are practically no data on targeting mutated p53 in OS. Still, some interesting results were recently obtained by Tang et al. [12], who used CRISPR-Cas to knock out mutated *TP53* in OS cell lines, affecting their oncogenic properties. Similar results were obtained with NSC59984, which accelerates mutant p53 degradation. Both strategies decreased the expression of anti-apoptotic proteins survivin and Bcl-2 in the two tested OS cell lines; however, as mentioned above, many OSs do not carry mutated p53, but rearrangements involving its promoter, and Tang et al. only tested two OS cell lines [12].

### 5.1. Gene Therapy Approaches

The *TP53* gene is also an important target for gene therapies [169]. Reduction of osteosarcoma cell proliferation, apoptosis induction, suppression of cell motility, and chemotherapy drug sensitivity enhancement as an effect of knocking out of mutant *TP53* was reported [10,11,12]. In the last 20 years, most clinical trials (Table S2) concerning p53 gene therapy were conducted in China and the United States.

Gendicine™ (rAd-p53) is the first gene-drug—recombinant human p53 adenovirus. In 2003, the China Food and Drug Administration (CFDA) approved it to treat head and neck squamous cell carcinoma when used in combination with radiotherapy. The recombinant human *TP53* gene is delivered into cancer cells by an adenovirus vector, and as a result, p53 protein is expressed. Gendicine is being used in China to treat patients with other advanced or unresectable tumors, including advanced lung cancer, liver cancer, gynecological tumors, and soft tissue sarcomas. Multimodal therapy with Gendicine and chemotherapy, radiotherapy, or other conventional treatment regimens demonstrated significantly higher response rates and progression-free survival than standard therapies alone [169]. The use of Gendicine in OS is to be evaluated.

At this point, alternative molecules that have been proven useful for redirecting adenoviral attachment and entry may be of potential interest in targeting OS, including fibroblast growth factor receptor, folate receptor, transferrin receptor, and vascular endothelial growth factor receptor [170]. Targeting the transferrin receptor was shown to be effective in OS. Nakase et al. studied liposome-p53 gene delivery targeted toward transferrin in vitro and in vivo and demonstrated reduced tumor growth of established xenografts [171].

### 5.2. Pharmacological Modulation of P53 Function

**Figure 3:**
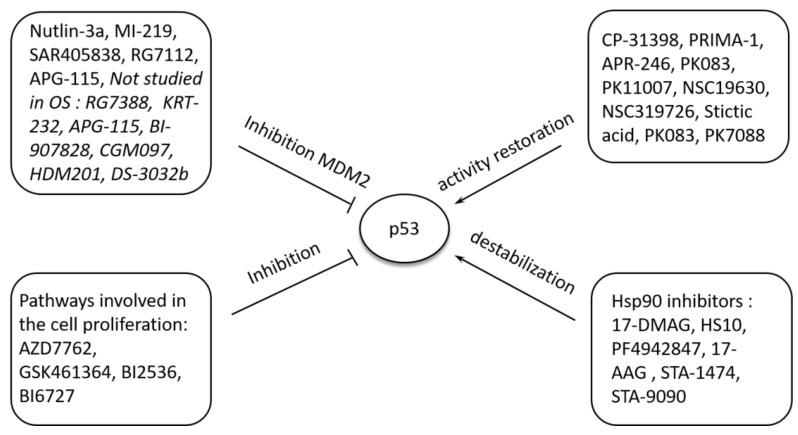
The main targets for pharmacological p53 modulation and selected molecules targeting p53-dependent pathways.

Pharmacological targeting of p53 is another approach used in the development of novel therapeutic strategies. There were few ways for p53 pharmacological modulation (Figure 3). The first method is the pharmacological inhibition of the principal ubiquitin ligase for p53, MDM2. One of the most broadly studied MDM2 antagonists is Nutlin-3a (RG7112), a molecule that acts through blockage of MDM2 in the p53-binding pocket, stabilizing the biologically active p53 protein [172]. Nutlin-3a was studied in preclinical studies on OS cell lines, and promising results showed that cells with wild-type p53 are sensitive to Nutlin-3a [173]. The clinical activity of Nutlin-3a was studied in an early phase clinical study of 20 patients with liposarcomas in a neoadjuvant setting. Unfortunately, unsatisfactory response rates were observed, with only one partial response obtained in a patient with *MDM2* amplification [174]. Interestingly, it was documented in an ex vivo tissue explant system of sarcoma tissue biopsies that *MDM2* status does not correlate with Nutlin-3a induced apoptosis [175]. An alternative target gene that could predict Nutlin-3a response was identified in this study: *GADD45A*, which, when not hypermethylated, was associated with the apoptotic response following Nutlin-3a treatment [175].

Since the development of Nutlin-3a, new generations of MDM2 inhibitors have been identified. MI-219 is an MDM2 inhibitor with higher affinity and selectivity than Nutlin-3a [176]; however, no data on its activity on OS in preclinical and clinical settings are available. The therapeutic effectiveness of another MDM2 small-molecule inhibitor, SAR405838, was assessed in in vivo and in vitro studies in dedifferentiated liposarcoma. Compared to other MDM2 inhibitors, Nutlin-3a and MI-219, treatment with SAR405838 resulted in similar antitumor activity [177]. Unfortunately, no objective responses were achieved in a phase I study of SAR405838 in patients with solid tumors [178]. In the subgroup of patients with de-differentiated liposarcoma, 22 of 31 patients (71%) achieved stable disease as the best response.

Another small molecule MDM2 inhibitor, RG7112, showed antitumor activity in a panel of different solid tumor cell lines with the strongest response in OS cells [179]. Moreover, RG7112 inhibited tumor growth in a dose-dependent manner in human OS xenografts with *MDM2* gene amplification and MDM2 protein overexpression [179].

APG-115, another small molecule MDM2 inhibitor, was tested in a phase I study of 21 patients with liposarcomas and other solid tumors, including one with OS. In the study, one partial response and 12 stable diseases were achieved [180]. Among these 13 patients, 12 had *TP53*-wild-type tumors, and 7 had concurrent *MDM2*-amplification. Other MDM2 antagonists included idasanutlin (RG7388), AMG-232 (KRT-232), APG-115, BI-907828, CGM097, siremadlin (HDM201), and milademetan (DS-3032b); however, they have not yet been studied in OS [181].

Another approach to target p53 function with pharmacological intervention is restoring wild-type p53 activity by promoting proper folding of mutant p53. Since the development of the first p53-reactivating compound, CP-31398, several mutant p53-targeting substances were identified, including PRIMA-1 and its methylated analog APR-246, PK083, PK11007, NSC19630, NSC319726, and others [70,182]. Despite numerous preclinical investigations, data on the antitumor activity of these compounds in OS are scarce. The compound PRIMA-1 was identified in a cellular screening for substances that suppressed the proliferation of Saos-2 osteosarcoma cells in a p53-dependent manner [183]. Stictic acid, a natural product identified as a high-affinity binder to the L1/S3 pockets, could reactivate p21 expression in R175H mutant OS cells in a dose-dependent manner more strongly than PRIMA-1 [184]. Moreover, APR-246, a methylated analog of PRIMA-1, demonstrated synergistic activity with a quinoline alkaloid camptothecin, the Saos-2 osteosarcoma cell line [185]. STIMA-1, a small molecule compound similar to CP-31398, induced p53-dependent apoptosis in Saos2-osteosarcoma cells [186]. Unfortunately, no clinical investigations on this type of approach were carried out in OS patients to date.

Moreover, reactivation of the *wt* p53 function can be achieved by targeting specific p53 mutations. The Y220C mutation was identified as the eighth most common *TP53* mutation [187]. Small-molecule stabilizers such as PK083 and PK7088 can bind to Y220C surface crevice [115,188], resulting in refolding into the *wt* p53 conformation. Suppression of other p53 mutants, including R175H and R273H, can be induced using P53R3, which has shown p53-dependent inhibition of glioma cell proliferation [189]. SCH529074, a small molecule activator of mutant p53, binds p53 DNA-Binding Domain (DBD) and demonstrated mutant-p53 reactivating abilities [190]. The compounds mentioned above have not yet been tested in preclinical or clinical settings in OS; however, they may have a potential role in OS treatment in the future.

Apart from restoring wild-type p53 activity, depletion of mutant p53 was studied as a possible approach to target p53 function in cancer [191]. Cholesterol-lowering drugs, statins, were hypothesized to suppress cancer cell growth by reducing mevalonate-5-phosphate, leading to CHIP ubiquitin ligase-mediated degradation of mutant p53. In the OS cell line, KHOS statins were shown to reduce mutant p53 levels and suppress cell proliferation [192].

A group of compounds that act through mutant p53 destabilization are heat shock protein 90 (Hsp90) inhibitors. Hsp90 causes the accumulation of mutant p53 by inactivating MDM2 and CHIP [193,194]. Treatment of undifferentiated pleomorphic sarcoma cells with Hsp90 inhibitor, 17-DMAG, led to decreased viability and invasiveness of the malignant cells [195]. Another Hsp90 inhibitor, HS10, showed synergistic activity with doxorubicin in OS cell lines [195]. PF4942847, a synthetic Hsp90 inhibitor, induced apoptosis, inhibited cell growth in a dose-dependent manner in an OS cell line, inhibited tumor growth, prolonged survival, and inhibited pulmonary metastases when administered orally in the OS mouse model [196]. Other Hsp90 inhibitors that showed potential antitumor activity in preclinical OS studies include 17-AAG [151], STA-1474 [152], STA-9090 (ganetespib) [197]. Ganetespib is a second-generation Hsp90 inhibitor with higher potency in degrading mutated p53 than the first-generation Hsp90 inhibitor 17-AAG [198]. Over 20 different Hsp90 inhibitors have been evaluated in clinical trials in various tumors; however, without effects sufficient for FDA approval [199]. Moreover, no clinical data on treatment with Hsp90 inhibitors in OS patients are available.

Another way to target p53 is treatment with substances that exclusively inhibit pathways involved in the proliferation of p53-mutated cells, including Chk1, polo-like kinase 1 (Plk1), and wee1 kinase (Wee1) [200]. AZD7762, a Chk1 inhibitor, induced cisplatin-mediated apoptosis in MNNG/HOS and Saos-2 osteosarcoma cell lines and caused inhibition of xenograft growth induced by cisplatin in an OS xenograft orthotopic model [201]. Treatment of U2OS, MG63, and SJSA osteosarcoma cell lines with GSK461364, a selective Plk1 inhibitor, resulted in mitotic arrest, apoptosis induction, enhanced cytotoxic effect when combined with paclitaxel [202]. Other inhibitors, BI2536 and BI6727, were also documented to have an antiproliferative effect on U2OS osteosarcoma cells mainly through enhanced MYC degradation [203]. The former substance was able to delay tumor growth in the OS xenograft mouse model [203].

Several Plk1 inhibitors are currently investigated in clinical trials in various cancer types, but none in OS patients [204]. Wee1 is another kinase that is crucial for proliferation in the p53-mutated cell. It controls the G2–M cell-cycle checkpoint and negatively regulates mitotic entry. Wee1 inhibition by a small molecule compound, PD0166285 in MG-63, U2OS, and Saos-2 osteosarcoma cell lines resulted in their sensitization to irradiation-induced cell death [205]. Another Wee1 inhibitor, MK-1775, enhanced the cytotoxic effect of gemcitabine in MG63, A673, U2OS, and HT-1080 osteosarcoma cell lines, OS-patient tumor explants, and a patient-derived mouse xenograft model. Interestingly, the effect of MK-1775 was independent of the p53 status of the cells [206]. Wee1 inhibitors were intensively studied in various tumors, including pancreatic, uterine, head and neck cancers, and hematologic malignancies [207]. To date, there are no data on the use of Wee1 inhibitors in OS patients.

NSC59984 is a small molecular compound that destabilizes mutant p53 and restores the *wt* p53 pathway via the activation of p73. At effective doses, it preferentially induces the death of tumor cells, stimulates p73 activity, and targets mutant p53 for degradation. This compound is nongenotoxic; thus, its clinical application could be possible. In contrast to p53, p73, a transcription factor with high structural and sequence homology top53 and has similar functions, is rarely deleted or mutated in human cancer. In mammalian cells under stress conditions, p73 is activated by complex signaling pathways that induce apoptosis and increase chemosensitivity. Many chemotherapeutic agents, such as camptothecin, etoposide, and cisplatin, up-regulate p73 expression. Mutant p53 inhibits p73 activation by binding to p73.

Additionally, NSC59984 induces mutant p53 protein degradation via MDM2-mediated ubiquitination and proteasomal degradation. Stabilization of mutant p53 results from its inability to interact with MDM2, an E3 ubiquitin ligase. It is unclear how mutant p53 and MDM2 are phosphorylated by signaling pathways stimulated by NSC59984 in mutant p53-expressing cancer cells. According to Fan Tang et al. (2019), NSC59984 strongly inhibits the expression of mutant p53 R156P in osteosarcomas. NSC59984 treatment results in mutant p53 degradation by MDM2-mediated ubiquitination and proteasomal degradation and restores TP53 pathway signaling via p73 activation. Administration of NSC59984 up-regulated the expression of p21, a potent CDK inhibitor that leads to cell-cycle arrest [12,208]. Fan Tang and et al. (2019) reported that the knockout of mutant *TP53* using CRISPR-Cas 9 increases the cytotoxic effect of doxorubicin in MDR osteosarcoma cells. Improvement of the anti-cancer activity of doxorubicin was observed after NSC59984 treatment in MDR osteosarcoma cells in vitro. Thus, a potential application of NSC59984 would be to reduce chemoresistance [12].

Association between p53 and IGF-1R receptors is not fully understood but is expected to impact OS cell viability. IGF-1 is considered one of the main factors in osteosarcoma pathogenesis, so modulating the IGF-IR pathway is an attractive anticancer treatment strategy in p53 mutated OS. PPP (Picropodophyllin, a member of the cyclolignan family, a new inhibitor of IGF-IR) inhibits IGF-IR expression in osteosarcoma cell lines. PPP was shown to be active against osteosarcoma cell lines resistant to conventional chemotherapy with doxorubicin, paclitaxel, and vincristine. PPP is also active against primary tumor cells from osteosarcoma patients [159,209,210].

## 6. Conclusions

After the introduction of doxorubicin, methotrexate, and cisplatin in the late 1970s, the 5-year overall survival of OS patients and therapeutic outcomes have remained essentially unchanged [211]. Currently, there is an urgent need to identify novel cellular targets for developing new therapeutic regimens because of limitations in the optimal treatment of OS patients [12]. Recent cooperative international prospective clinical trials have attempted to intensify treatments or modulate immune responses but have had limited success without improving drug efficacy or toxicity [212,213].

Because a significant fraction of human cancers has mutations in the *TP53* gene, the wild-type *TP53* gene has become an important target for novel cancer gene therapy [169]. The *TP53* gene encodes an important transcription factor (p53) with a tumor suppressor role. Mutated *TP53* seems to play a critical role in maintaining cell proliferation and growth in OSs. In response to cellular stress, it activates various pathways that induce cell-cycle arrest, apoptosis, cell senescence, DNA repair, or changes in metabolism. Moreover, p53 plays a regulatory role in ontogenesis, myogenesis, angiogenesis [214]. p53 is also involved in activating several genes encoding apoptotic proteins (like PIG, BAX, Fas/Apo1, Killer/DR5, PUMA, or Noxa) [27,28,29], inducing pluripotency [215], regulating various metabolic pathways (reduction of glucose uptake, repression of transcription of *GLUT1* and *GLUT4* transporters genes) [54], or modulating inflammatory reactions [59,60,216]. Altered p53 may also contribute to drug resistance [64,106].

The presence of a mutation in the *TP53* gene may exert three possible effects: deprivation of the ‘guardian’ p53 function, overcontrolling its co-expressed wild-type form (the dominant-negative effect), or gain-of-function [72]. However, these possibilities are not necessarily exclusive. Mutations in the *TP53* gene frequently occur in Li–Fraumeni syndrome. Patients suffering from LFS develop sarcomas most frequently (32.7%). *TP53* mutations are also commonly detected in OS specimens; current studies estimate that these mutations are present in 47–90% of cases. Importantly, somatic *TP53* mutations are an unfavorable prognostic marker in OS patients [120].

The p53 protein is considered a challenging target for cancer therapy. In particular, the challenges are associated with numerous different mutations and their varied effects on the p53 protein. Even in the presence of the wild-type protein, the mutated protein may affect the structure of the whole tetramer [77]. Currently, the most promising approach is gene therapy. The China Food and Drug Administration (CFDA) approved the first anti-tumor gene therapy drug rAd-p53 (recombinant human p53 adenovirus), Gendicine™. However, it was approved for treating head and neck squamous cell carcinoma (HNSCC) in combination with radiotherapy, and the data on its therapeutic potential in OS is not available. Further clinical trials on *TP53*-targeted gene therapy approaches are also conducted in academic centers outside China.

Another approach is pharmacological p53 modulation. Several agents are in an early phase of development. One example could be the pharmacological inhibition of the principal ubiquitin ligase for p53, MDM2, with Nutlin-3a (RG7112) [172] or SAR405838 [177]. Some pharmacological interventions, e.g., CP-31398, attempt to restore the wild-type p53 activity by promoting proper folding of mutant p53 [70,182]. Another interesting approach is the administration of heat shock protein 90 (Hsp90) inhibitors (Hsp90 causes accumulation of mutant p53 by inactivating MDM2 and CHIP [193,194]). Unfortunately, the data on the possible application of p53-targeted pharmacological interventions in OS are currently scarce.

## Figures and Tables

**Table 1:** Overview of animal models of osteosarcoma.

Species	Mutations	Malignancies	References
Mouse	Trp53 knockout	Spindle cell sarcomas and pleomorphic sarcomas develop if *Trp53* is inactivated by Cre-loxP-mediated recombination	[135]
Mouse	p53 R172H and p53 R270H mutations	The spectrum of tumors that develop in mice depends on the various mutant alleles in the model. p. R270H (p. R273H in humans) results in a high frequency of carcinomas, whereas the ‘structural’ mutant p. R172H (p. R175H in humans) predominantly results in OSs.	[137]
Rat (Dark Agouti)	*Tp53* knockout	Homozygotes develop angiosarcomas and lymphomas, and their lifespan is limited to 6 months. Heterozygotes live longer (up to 12 months) and develop a broader spectrum of tumors; however, angiosarcomas and lymphomas are also the most common, and OSs are rare	[140,141]
Rat *(*Wistar*)*	Tp53C273X/C273X mutation.	Homozygotes develop mostly angiosarcomas, while heterozygotes develop OSs and less frequently angiosarcomas and B-cell lymphomas	[139,145].
Rat (Sprague Dawley)	*Tp53* knockout	Mostly sarcomas. The spectrum of tumors seems to be generally broader than in the Wistar knockout rats. OS is the most frequently observed type of tumor in homozygotes, but still, it constitutes only 18% of all tumors	[145]
Zebrafish (*Danio rerio*)	mutation p53 I166T	Both heterozygous and mutant fish developed soft tissue sarcomas with high penetrance but most frequently, spindle cell tumors	[146].
Zebrafish (*Danio rerio*)	*Tp53* knockout	Wide range of tumors, including angiosarcomas, malignant peripheral nerve sheath tumors, germ cell tumors, or leukemias	[147]
Pet dogs	Spontaneous *Tp53* mutations	Tp53 mutations were found in 40% of OSs	[151,152]

## Supplementary Materials

The following are available online at https://www.mdpi.com/article/10.3390/cancers13174284/s1, Table S1: List of missense mutations in the *TP53* gene found in osteosarcoma derived patient samples, Table S2: Overview of clinical trials retrieved from ClinicalTrials.gov (accessed on 22 October 2020) and EU Clinical Trials Register.

## References

[1] Rutkowski P., Świtaj T. (2018). Bone sarcomas. Oncol. Clin. Pract..

[2] Wang H., Miao R., Jacobson A., Goldberg S., Harmon D.C., Cote G., Hornicek F.J., Raskin K., Nielsen G., DeLaney T.F. (2017). Osteosarcoma prognostic nomograms for predicting the 10-year probability of mortality and recurrence. J. Clin. Oncol..

[3] Spalek M.J., Poleszczuk J., Czarnecka A.M., Dudzisz-Sledz M., Napieralska A., Matysiakiewicz J., Chojnacka M., Raciborska A., Sztuder A., Maciejczyk A. (2021). Radiotherapy in the Management of Pediatric and Adult Osteosarcomas: A Multi-Institutional Cohort Analysis. Cells.

[4] Casali P.G., Bielack S., Abecassis N., Aro H.T., Bauer S., Biagini R., Bonvalot S., Boukovinas I., Bovee J., Brennan B. (2018). Bone sarcomas: ESMO-PaedCan-EURACAN Clinical Practice Guidelines for diagnosis, treatment and follow-up. Ann. Oncol..

[5] Picci P. (2007). Osteosarcoma (osteogenic sarcoma). Orphanet. J. Rare Dis..

[6] Grignani G., Palmerini E., Ferraresi V., Asaftei S.D., D’Ambrosio L., Pignochino Y., Nuzzo A., Gambarotti M., Marchesi E., Biagini R. (2014). A nonrandomized phase II trial of sorafenib (S) and everolimus (E) in unresectable metastatic osteosarcoma (OST) patients (pts) relapsed after standard chemotherapy. J. Clin. Oncol..

[7] Xie L., Guo W., Xu J., Sun X., Liu K., Zheng B., Ren T., Huang Y., Tang X., Yan T. (2019). Apatinib plus camrelizumab (SHR-1210) for unresectable high-grade osteosarcoma (APFAO) progressing after chemotherapy: A prospective, open label, phase II trial. J. Clin. Oncol..

[8] Smrke A., Anderson P.M., Gulia A., Gennatas S., Huang P.H., Jones R.L. (2021). Future Directions in the Treatment of Osteosarcoma. Cells.

[9] Bishop M.W., Janeway K.A., Gorlick R. (2016). Future directions in the treatment of osteosarcoma. Curr. Opin. Pediatr..

[10] Kang N., Wang Y., Guo S., Ou Y., Wang G., Chen J., Li D., Zhan Q. (2018). Mutant TP53 G245C and R273H promote cellular malignancy in esophageal squamous cell carcinoma. BMC Cell Biol..

[11] Doyle B., Morton J.P., Delaney D.W., Ridgway R.A., Wilkins J.A., Sansom O.J. (2010). p53 mutation and loss have different effects on tumourigenesis in a novel mouse model of pleomorphic rhabdomyosarcoma. J. Pathol..

[12] Tang F., Min L., Seebacher N.A., Li X., Zhou Y., Hornicek F.J., Wei Y., Tu C., Duan Z. (2019). Targeting mutant TP53 as a potential therapeutic strategy for the treatment of osteosarcoma. J. Orthop. Res. Off. Publ. Orthop. Res. Soc..

[13] Sanz G., Singh M., Peuget S., Selivanova G. (2019). Inhibition of p53 inhibitors: Progress, challenges and perspectives. J. Mol. Cell Biol..

[14] Zhao X., Wu Q., Gong X., Liu J., Ma Y. (2021). Osteosarcoma: A review of current and future therapeutic approaches. Biomed. Eng. Online.

[15] Czarnecka A.M., Synoradzki K., Firlej W., Bartnik E., Sobczuk P., Fiedorowicz M., Grieb P., Rutkowski P. (2020). Molecular Biology of Osteosarcoma. Cancers.

[16] Vieler M., Sanyal S. (2018). p53 Isoforms and Their Implications in Cancer. Cancers.

[17] Bourdon J.-C., Fernandes K., Murray-Zmijewski F., Liu G., Diot A., Xirodimas D.P., Saville M.K., Lane D.P. (2005). p53 isoforms can regulate p53 transcriptional activity. Genes Dev..

[18] Courtois S., Verhaegh G., North S., Luciani M.-G., Lassus P., Hibner U., Oren M., Hainaut P. (2002). ΔN-p53, a natural isoform of p53 lacking the first transactivation domain, counteracts growth suppression by wild-type p53. Oncogene.

[19] Toledo F., Wahl G.M. (2006). Regulating the p53 pathway: In vitro hypotheses, in vivo veritas. Nat. Rev. Cancer.

[20] Bourdon J.C. (2007). p53 and its isoforms in cancer. Br. J. Cancer.

[21] Sullivan K.D., Galbraith M.D., Andrysik Z., Espinosa J.M. (2018). Mechanisms of transcriptional regulation by p53. Cell Death Differ..

[22] Tan Y.S., Mhoumadi Y., Verma C.S. (2019). Roles of computational modelling in understanding p53 structure, biology, and its therapeutic targeting. J. Mol. Cell Biol..

[23] Raj N., Attardi L.D. (2017). The Transactivation Domains of the p53 Protein. Cold Spring Harb. Perspect. Med..

[24] O’Keefe K., Li H., Zhang Y. (2003). Nucleocytoplasmic shuttling of p53 is essential for MDM2-mediated cytoplasmic degradation but not ubiquitination. Mol. Cell Biol..

[25] Vogelstein B., Lane D., Levine A.J. (2000). Surfing the p53 network. Nature.

[26] Shi T., Polderman P.E., Burgering B.M.T., Dansen T.B. (2020). DNA damage and oxidizing conditions activate p53 through differential upstream signaling pathways. bioRxiv.

[27] el-Deiry W.S. (1998). Regulation of p53 downstream genes. Semin. Cancer Biol..

[28] Sax J.K., El-Deiry W.S. (2003). p53 downstream targets and chemosensitivity. Cell Death Differ..

[29] Yu J., Zhang L., Hwang P.M., Rago C., Kinzler K.W., Vogelstein B. (1999). Identification and classification of p53-regulated genes. Proc. Natl. Acad. Sci. USA.

[30] Wu G.S., Burns T.F., McDonald E.R., Meng R.D., Kao G., Muschel R., Yen T., El-Deiry W.S. (1999). Induction of the TRAIL receptor KILLER/DR5 in p53-dependent apoptosis but not growth arrest. Oncogene.

[31] Tait S.W.G., Green D.R. (2010). Mitochondria and cell death: Outer membrane permeabilization and beyond. Nat. Rev. Mol. Cell Biol..

[32] Yu J., Yue W., Wu B., Zhang L. (2006). PUMA sensitizes lung cancer cells to chemotherapeutic agents and irradiation. Clin. Cancer Res..

[33] Oda E., Ohki R., Murasawa H., Nemoto J., Shibue T., Yamashita T., Tokino T., Taniguchi T., Tanaka N. (2000). Noxa, a BH3-Only Member of the Bcl-2 Family and Candidate Mediator of p53-Induced Apoptosis. Science.

[34] Nakano K., Vousden K.H. (2001). PUMA, a Novel Proapoptotic Gene, Is Induced by p53. Mol. Cell.

[35] Michalak E.M., Villunger A., Adams J.M., Strasser A. (2008). In several cell types tumour suppressor p53 induces apoptosis largely via Puma but Noxa can contribute. Cell Death Differ..

[36] Ding Y., Wang Y., Chen J., Hu Y., Cao Z., Ren P., Zhang Y. (2014). p21 overexpression sensitizes osteosarcoma U2OS cells to cisplatin via evoking caspase-3 and Bax/Bcl-2 cascade. Tumour Biol. J. Int. Soc. Oncodev. Biol. Med..

[37] Crighton D., Wilkinson S., O’Prey J., Syed N., Smith P., Harrison P.R., Gasco M., Garrone O., Crook T., Ryan K.M. (2006). DRAM, a p53-Induced Modulator of Autophagy, Is Critical for Apoptosis. Cell.

[38] Tasdemir E., Maiuri M.C., Galluzzi L., Vitale I., Djavaheri-Mergny M., D’Amelio M., Criollo A., Morselli E., Zhu C., Harper F. (2008). Regulation of autophagy by cytoplasmic p53. Nat. Cell Biol..

[39] Du Y., Yang D., Li L., Luo G., Li T., Fan X., Wang Q., Zhang X., Wang Y., Le W. (2009). An insight into the mechanistic role of p53-mediated autophagy induction in response to proteasomal inhibition-induced neurotoxicity. Autophagy.

[40] Jiang L., Kon N., Li T., Wang S.-J., Su T., Hibshoosh H., Baer R., Gu W. (2015). Ferroptosis as a p53-mediated activity during tumour suppression. Nature.

[41] Gartel A.L., Tyner A.L. (2002). The role of the cyclin-dependent kinase inhibitor p21 in apoptosis. Mol. Cancer Ther..

[42] Waldman T., Kinzler Kw Fau-Vogelstein B., Vogelstein B. (1995). p21 is necessary for the p53-mediated G1 arrest in human cancer cells. Cancer Res..

[43] Yosef R., Pilpel N., Papismadov N., Gal H., Ovadya Y., Vadai E., Miller S., Porat Z., Ben-Dor S., Krizhanovsky V. (2017). p21 maintains senescent cell viability under persistent DNA damage response by restraining JNK and caspase signaling. EMBO J..

[44] Zhang Y., Gao Y., Zhang G., Huang S., Dong Z., Kong C., Su D., Du J., Zhu S., Liang Q. (2011). DNMT3a plays a role in switches between doxorubicin-induced senescence and apoptosis of colorectal cancer cells. Int. J. Cancer.

[45] Gartel A.L., Tyner A.L. (1999). Transcriptional regulation of the p21((WAF1/CIP1)) gene. Exp. Cell Res..

[46] Holzer K., Drucker E., Roessler S., Dauch D., Heinzmann F., Waldburger N., Eiteneuer E.M., Herpel E., Breuhahn K., Zender L. (2017). Proteomic Analysis Reveals GMP Synthetase as p53 Repression Target in Liver Cancer. Am. J. Pathol..

[47] Mijit M., Caracciolo V., Melillo A., Amicarelli F., Giordano A. (2020). Role of p53 in the Regulation of Cellular Senescence. Biomolecules.

[48] Tang Y., Yang C., Guo Z., Fu Y., Yu X., Liu B., Zhou H., Wang J., Li W., Pang Q. (2017). P16 protein expression as a useful predictive biomarker for neoadjuvant chemotherapy response in patients with high-grade osteosarcoma: A systematic meta-analysis under guideline of PRISMA. Medicine.

[49] Beauséjour C.M., Krtolica A., Galimi F., Narita M., Lowe S.W., Yaswen P., Campisi J. (2003). Reversal of human cellular senescence: Roles of the p53 and p16 pathways. EMBO J..

[50] Kawamura T., Suzuki J., Wang Y.V., Menendez S., Morera L.B., Raya A., Wahl G.M., Izpisúa Belmonte J.C. (2009). Linking the p53 tumour suppressor pathway to somatic cell reprogramming. Nature.

[51] Zhao Y., Yin X., Qin H., Zhu F., Liu H., Yang W., Zhang Q., Xiang C., Hou P., Song Z. (2008). Two Supporting Factors Greatly Improve the Efficiency of Human iPSC Generation. Cell Stem Cell.

[52] Yi L., Lu C., Hu W., Sun Y., Levine A.J. (2012). Multiple roles of p53-related pathways in somatic cell reprogramming and stem cell differentiation. Cancer Res..

[53] Sarig R., Rivlin N., Brosh R., Bornstein C., Kamer I., Ezra O., Molchadsky A., Goldfinger N., Brenner O., Rotter V. (2010). Mutant p53 facilitates somatic cell reprogramming and augments the malignant potential of reprogrammed cells. J. Exp. Med..

[54] Schwartzenberg-Bar-Yoseph F., Armoni M., Fau-Karnieli E., Karnieli E. (2004). The tumor suppressor p53 down-regulates glucose transporters GLUT1 and GLUT4 gene expression. Cancer Res..

[55] Bensaad K., Tsuruta A., Selak M.A., Vidal M.N., Nakano K., Bartrons R., Gottlieb E., Vousden K.H. (2006). TIGAR, a p53-inducible regulator of glycolysis and apoptosis. Cell.

[56] Ros S., Flöter J., Kaymak I., Da Costa C., Houddane A., Dubuis S., Griffiths B., Mitter R., Walz S., Blake S. (2017). 6-Phosphofructo-2-kinase/fructose-2,6-biphosphatase 4 is essential for p53-null cancer cells. Oncogene.

[57] Franklin D.A., He Y., Leslie P.L., Tikunov A.P., Fenger N., Macdonald J.M., Zhang Y. (2016). p53 coordinates DNA repair with nucleotide synthesis by suppressing PFKFB3 expression and promoting the pentose phosphate pathway. Sci. Rep..

[58] Matoba S., Kang J.G., Fau-Patino W.D., Patino Wd Fau-Wragg A., Wragg A., Fau-Boehm M., Boehm M., Fau-Gavrilova O., Gavrilova O., Fau-Hurley P.J. (2006). p53 regulates mitochondrial respiration. Science.

[59] Xue W., Zender L., Miething C., Dickins R.A., Hernando E., Krizhanovsky V., Cordon-Cardo C., Lowe S.W. (2007). Senescence and tumour clearance is triggered by p53 restoration in murine liver carcinomas. Nature.

[60] Yeudall W.A., Vaughan C.A., Miyazaki H., Ramamoorthy M., Choi M.Y., Chapman C.G., Wang H., Black E., Bulysheva A.A., Deb S.P. (2021). Gain-of-function mutant p53 upregulates CXC chemokines and enhances cell migration. Carcinogenesis.

[61] Li B., Wang Z., Wu H., Xue M., Lin P., Wang S., Lin N., Huang X., Pan W., Liu M. (2018). Epigenetic Regulation of CXCL12 Plays a Critical Role in Mediating Tumor Progression and the Immune Response In Osteosarcoma. Cancer Res..

[62] Rahnamoun H., Lu H., Duttke S.H., Benner C., Glass C.A.-O., Lauberth S.M. (2017). Mutant p53 shapes the enhancer landscape of cancer cells in response to chronic immune signaling. Nat. Commun..

[63] Zhou J., Liu T., Wang W. (2018). Prognostic significance of matrix metalloproteinase 9 expression in osteosarcoma: A meta-analysis of 16 studies. Medicine.

[64] Martelli L., Di Mario F., Botti P., Ragazzi E., Martelli M., Kelland L. (2007). Accumulation, platinum–DNA adduct formation and cytotoxicity of cisplatin, oxaliplatin and satraplatin in sensitive and resistant human osteosarcoma cell lines, characterized by p53 wild-type status. Biochem. Pharmacol..

[65] Goto A., Kanda H., Ishikawa Y., Matsumoto S., Kawaguchi N., Machinami R., Kato Y., Kitagawa T. (1998). Association of loss of heterozygosity at the p53 locus with chemoresistance in osteosarcomas. Jpn. J. Cancer Res..

[66] Huang X., Zhang S., Qi H., Wang Z., Chen H.-W., Shao J., Shen J. (2015). JMJD5 interacts with p53 and negatively regulates p53 function in control of cell cycle and proliferation. Biochim. Biophys. Acta (BBA)-Mol. Cell Res..

[67] Midgley C.A., Desterro J.M., Saville M.K., Howard S., Sparks A., Hay R.T., Lane D.P. (2000). An N-terminal p14ARF peptide blocks Mdm2-dependent ubiquitination in vitro and can activate p53 in vivo. Oncogene.

[68] Pomerantz J., Schreiber-Agus N., Liégeois N.J., Silverman A., Alland L., Chin L., Potes J., Chen K., Orlow I., Lee H.-W. (1998). The *Ink4a* Tumor Suppressor Gene Product, p19^Arf^, Interacts with MDM2 and Neutralizes MDM2′s Inhibition of p53. Cell.

[69] Hainaut P., Pfeifer G.P. (2016). Somatic TP53 Mutations in the Era of Genome Sequencing. Cold Spring Harb. Perspect. Med..

[70] Bykov V.J.N., Eriksson S.E., Bianchi J., Wiman K.G. (2018). Targeting mutant p53 for efficient cancer therapy. Nat. Rev. Cancer.

[71] Blandino G., Di Agostino S. (2018). New therapeutic strategies to treat human cancers expressing mutant p53 proteins. J. Exp. Clin. Cancer Res. CR.

[72] Oren M., Rotter V. (2010). Mutant p53 gain-of-function in cancer. Cold Spring Harb Perspect Biol..

[73] Liu D.P., Song H., Xu Y. (2010). A common gain of function of p53 cancer mutants in inducing genetic instability. Oncogene.

[74] Willis A., Jung E.J., Wakefield T., Chen X. (2004). Mutant p53 exerts a dominant negative effect by preventing wild-type p53 from binding to the promoter of its target genes. Oncogene.

[75] Cho Y., Gorina S., Jeffrey P.D., Pavletich N.P. (1994). Crystal structure of a p53 tumor suppressor-DNA complex: Understanding tumorigenic mutations. Science.

[76] Joerger A.C., Ang H.C., Veprintsev D.B., Blair C.M., Fersht A.R. (2005). Structures of p53 cancer mutants and mechanism of rescue by second-site suppressor mutations. J. Biol. Chem..

[77] Levine A.J. (2019). Targeting Therapies for the p53 Protein in Cancer Treatments. Ann. Rev. Cancer Biol..

[78] Bouaoun L., Sonkin D., Ardin M., Hollstein M., Byrnes G., Zavadil J., Olivier M. (2016). TP53 Variations in Human Cancers: New Lessons from the IARC TP53 Database and Genomics Data. Hum. Mutat..

[79] Petitjean A., Mathe E., Kato S., Ishioka C., Tavtigian S.V., Hainaut P., Olivier M. (2007). Impact of mutant p53 functional properties on TP53 mutation patterns and tumor phenotype: Lessons from recent developments in the IARC TP53 database. Hum. Mutat.

[80] Behjati S., Tarpey P.S., Haase K., Ye H., Young M.D., Alexandrov L.B., Farndon S.J., Collord G., Wedge D.C., Martincorena I. (2017). Recurrent mutation of IGF signalling genes and distinct patterns of genomic rearrangement in osteosarcoma. Nat. Commun..

[81] Gokgoz N., Wunder J.S., Mousses S., Eskandarian S., Bell R.S., Andrulis I.L. (2001). Comparison of p53 mutations in patients with localized osteosarcoma and metastatic osteosarcoma. Cancer.

[82] Mirabello L., Yeager M., Mai P.L., Gastier-Foster J.M., Gorlick R., Khanna C., Patiño-Garcia A., Sierrasesúmaga L., Lecanda F., Andrulis I.L. (2015). Germline TP53 variants and susceptibility to osteosarcoma. J. Natl. Cancer Inst..

[83] Perry J.A., Kiezun A., Tonzi P., Van Allen E.M., Carter S.L., Baca S.C., Cowley G.S., Bhatt A.S., Rheinbay E., Pedamallu C.S. (2014). Complementary genomic approaches highlight the PI3K/mTOR pathway as a common vulnerability in osteosarcoma. Proc. Natl. Acad. Sci. USA.

[84] Kovac M., Blattmann C., Ribi S., Smida J., Mueller N.S., Engert F., Castro-Giner F., Weischenfeldt J., Kovacova M., Krieg A. (2015). Exome sequencing of osteosarcoma reveals mutation signatures reminiscent of BRCA deficiency. Nat. Commun..

[85] Bousquet M., Noirot C., Accadbled F., Sales de Gauzy J., Castex M.P., Brousset P., Gomez-Brouchet A. (2016). Whole-exome sequencing in osteosarcoma reveals important heterogeneity of genetic alterations. Ann. Oncol..

[86] Overholtzer M., Rao P.H., Favis R., Lu X.Y., Elowitz M.B., Barany F., Ladanyi M., Gorlick R., Levine A.J. (2003). The presence of p53 mutations in human osteosarcomas correlates with high levels of genomic instability. Proc. Natl. Acad. Sci. USA.

[87] Toguchida J., Yamaguchi T., Ritchie B., Beauchamp R.L., Dayton S.H., Herrera G.E., Yamamuro T., Kotoura Y., Sasaki M.S., Little J.B. (1992). Mutation spectrum of the p53 gene in bone and soft tissue sarcomas. Cancer Res..

[88] Patino-Garcia A., Sierrasesumaga L. (1997). Analysis of the p16INK4 and TP53 tumor suppressor genes in bone sarcoma pediatric patients. Cancer Genet. Cytogenet..

[89] Chen X., Bahrami A., Pappo A., Easton J., Dalton J., Hedlund E., Ellison D., Shurtleff S., Wu G., Wei L. (2014). Recurrent somatic structural variations contribute to tumorigenesis in pediatric osteosarcoma. Cell Rep..

[90] Miller C.W., Aslo A., Won A., Tan M., Lampkin B., Koeffler H.P. (1996). Alterations of the p53, Rb and MDM2 genes in osteosarcoma. J. Cancer Res. Clin. Oncol..

[91] Wadayama B., Toguchida J., Yamaguchi T., Sasaki M.S., Yamamuro T. (1993). p53 expression and its relationship to DNA alterations in bone and soft tissue sarcomas. Br. J. Cancer.

[92] Mousses S., McAuley L., Bell R.S., Kandel R., Andrulis I.L. (1996). Molecular and immunohistochemical identification of p53 alterations in bone and soft tissue sarcomas. Mod. Pathol..

[93] Lorenz S., Barøy T., Sun J., Nome T., Vodák D., Bryne J.C., Håkelien A.M., Fernandez-Cuesta L., Möhlendick B., Rieder H. (2016). Unscrambling the genomic chaos of osteosarcoma reveals extensive transcript fusion, recurrent rearrangements and frequent novel TP53 aberrations. Oncotarget.

[94] Cerami E., Gao J., Dogrusoz U., Gross B.E., Sumer S.O., Aksoy B.A., Jacobsen A., Byrne C.J., Heuer M.L., Larsson E. (2012). The cBio cancer genomics portal: An open platform for exploring multidimensional cancer genomics data. Cancer Discov..

[95] Gao J., Aksoy B.A., Dogrusoz U., Dresdner G., Gross B., Sumer S.O., Sun Y., Jacobsen A., Sinha R., Larsson E. (2013). Integrative analysis of complex cancer genomics and clinical profiles using the cBioPortal. Sci. Signal..

[96] Srivastava S., Zou Z., Pirollo K., Blattner W., Chang E.H. (1990). Germ-line transmission of a mutated p53 gene in a cancer-prone family with Li-Fraumeni syndrome. Nature.

[97] Malkin D., Li F.P., Strong L.C., Fraumeni J.F., Nelson C.E., Kim D.H., Kassel J., Gryka M.A., Bischoff F.Z., Tainsky M.A. (1990). Germ line p53 mutations in a familial syndrome of breast cancer, sarcomas, and other neoplasms. Science.

[98] Levine A.J. (2020). p53: 800 million years of evolution and 40 years of discovery. Nat. Rev. Cancer.

[99] Birch J.M., Hartley A.L., Tricker K.J., Prosser J., Condie A., Kelsey A.M., Harris M., Jones P.H., Binchy A., Crowther D. (1994). Prevalence and diversity of constitutional mutations in the p53 gene among 21 Li-Fraumeni families. Cancer Res..

[100] Li F.P., Fraumeni J.F., Mulvihill J.J., Blattner W.A., Dreyfus M.G., Tucker M.A., Miller R.W. (1988). A cancer family syndrome in twenty-four kindreds. Cancer Res..

[101] Chompret A., Abel A., Stoppa-Lyonnet D., Brugieres L., Pagès S., Feunteun J., Bonaïti-Pellié C.A. (2001). Sensitivity and predictive value of criteria for p53 germline mutation screening. J. Med. Genet..

[102] Bougeard G., Sesboüé R., Baert-Desurmont S., Vasseur S., Martin C., Tinat J., Brugières L., Chompret A., Paillerets B.B.-D., Stoppa-Lyonnet D. (2008). Molecular basis of the Li–Fraumeni syndrome: An update from the French LFS families. J. Med. Genet..

[103] Jennis M., Kung C.P., Basu S., Budina-Kolomets A., Leu J.I., Khaku S., Scott J.P., Cai K.Q., Campbell M.R., Porter D.K. (2016). An African-specific polymorphism in the TP53 gene impairs p53 tumor suppressor function in a mouse model. Genes Dev..

[104] Kang H.J., Chun S.M., Kim K.R., Sohn I., Sung C.O. (2013). Clinical relevance of gain-of-function mutations of p53 in high-grade serous ovarian carcinoma. PLoS ONE.

[105] Dittmer D., Pati S., Zambetti G., Chu S., Teresky A.K., Moore M., Finlay C., Levine A.J. (1993). Gain of function mutations in p53. Nat. Genet..

[106] Blandino G., Levine A.J., Oren M. (1999). Mutant p53 gain of function: Differential effects of different p53 mutants on resistance of cultured cells to chemotherapy. Oncogene.

[107] Tsang W.-P., Ho F.Y.F., Fung K.-P., Kong S.-K., Kwok T.-T. (2005). p53-R175H mutant gains new function in regulation of doxorubicin-induced apoptosis. Int. J. Cancer.

[108] Wong R.P.C., Tsang W.P., Chau P.Y., Co N.N., Tsang T.Y., Kwok T.T. (2007). p53-R273H gains new function in induction of drug resistance through down-regulation of procaspase-3. Mol. Cancer Therapeut..

[109] Zhang Y., Hu Q., Li G., Li L., Liang S., Zhang Y., Liu J., Fan Z., Li L., Zhou B. (2018). ONZIN Upregulation by Mutant p53 Contributes to Osteosarcoma Metastasis Through the CXCL5-MAPK Signaling Pathway. Cell. Physiol. Biochem. Int. J. Exp. Cell. Physiol. Biochem. Pharmacol..

[110] Xiong S., Tu H., Kollareddy M., Pant V., Li Q., Zhang Y., Jackson J.G., Suh Y.A., Elizondo-Fraire A.C., Yang P. (2014). Pla2g16 phospholipase mediates gain-of-function activities of mutant p53. Proc. Natl. Acad. Sci. USA.

[111] Lang G.A., Iwakuma T., Suh Y.A., Liu G., Rao V.A., Parant J.M., Valentin-Vega Y.A., Terzian T., Caldwell L.C., Strong L.C. (2004). Gain of function of a p53 hot spot mutation in a mouse model of Li-Fraumeni syndrome. Cell.

[112] Pourebrahim R., Zhang Y., Liu B., Gao R., Xiong S., Lin P.P., McArthur M.J., Ostrowski M.C., Lozano G. (2017). Integrative genome analysis of somatic p53 mutant osteosarcomas identifies Ets2-dependent regulation of small nucleolar RNAs by mutant p53 protein. Genes Dev..

[113] Joerger A.C., Ang H.C., Fersht A.R. (2006). Structural basis for understanding oncogenic p53 mutations and designing rescue drugs. Proc. Natl. Acad. Sci. USA.

[114] Jordan J.J., Inga A., Conway K., Edmiston S., Carey L.A., Wu L., Resnick M.A. (2010). Altered-function p53 missense mutations identified in breast cancers can have subtle effects on transactivation. Mol. Cancer Res..

[115] Boeckler F.M., Joerger A.C., Jaggi G., Rutherford T.J., Veprintsev D.B., Fersht A.R. (2008). Targeted rescue of a destabilized mutant of p53 by an in silico screened drug. Proc. Natl. Acad. Sci. USA.

[116] Tanaka N., Zhao M., Tang L., Patel A.A., Xi Q., Van H.T., Takahashi H., Osman A.A., Zhang J., Wang J. (2018). Gain-of-function mutant p53 promotes the oncogenic potential of head and neck squamous cell carcinoma cells by targeting the transcription factors FOXO3a and FOXM1. Oncogene.

[117] Neskey D.M., Osman A.A., Ow T.J., Katsonis P., McDonald T., Hicks S.C., Hsu T.K., Pickering C.R., Ward A., Patel A. (2015). Evolutionary Action Score of TP53 Identifies High-Risk Mutations Associated with Decreased Survival and Increased Distant Metastases in Head and Neck Cancer. Cancer Res..

[118] Pintus S.S., Ivanisenko N.V., Demenkov P.S., Ivanisenko T.V., Ramachandran S., Kolchanov N.A., Ivanisenko V.A. (2013). The substitutions G245C and G245D in the Zn(2+)-binding pocket of the p53 protein result in differences of conformational flexibility of the DNA-binding domain. J. Biomol. Struct. Dyn..

[119] Franceschini N., Lam S.W., Cleton-Jansen A.M., Bovée J. (2020). What’s new in bone forming tumours of the skeleton?. Virchows Arch. Int. J. Pathol..

[120] Chen Z., Guo J., Zhang K., Guo Y. (2016). TP53 Mutations and Survival in Osteosarcoma Patients: A Meta-Analysis of Published Data. Dis. Mark..

[121] Simpson D.A., Livanos E., Heffernan T.P., Kaufmann W.K. (2005). Telomerase expression is sufficient for chromosomal integrity in cells lacking p53 dependent G1 checkpoint function. J. Carcinog..

[122] Slováčkova J., Grochova D., Navratilova J., Smarda J., Smardova J. (2010). Transactivation by temperature-dependent p53 mutants in yeast and human cells. Cell Cycle.

[123] Grochova D., Vankova J., Damborsky J., Ravcukova B., Smarda J., Vojtesek B., Smardova J. (2008). Analysis of transactivation capability and conformation of p53 temperature-dependent mutants and their reactivation by amifostine in yeast. Oncogene.

[124] Schärer E., Iggo R. (1992). Mammalian p53 can function as a transcription factor in yeast. Nucleic Acids Res..

[125] Kato S., Han S.-Y., Liu W., Otsuka K., Shibata H., Kanamaru R., Ishioka C. (2003). Understanding the function–structure and function–mutation relationships of p53 tumor suppressor protein by high-resolution missense mutation analysis. Proc. Natl. Acad. Sci. USA.

[126] Achatz M.I., Olivier M., Le Calvez F., Martel-Planche G., Lopes A., Rossi B.M., Ashton-Prolla P., Giugliani R., Palmero E.I., Vargas F.R. (2007). The TP53 mutation, R337H, is associated with Li-Fraumeni and Li-Fraumeni-like syndromes in Brazilian families. Cancer Lett..

[127] Giacomazzi J., Graudenz M.S., Osorio C.A.B.T., Koehler-Santos P., Palmero E.I., Zagonel-Oliveira M., Michelli R.A.D., Neto C.S., Fernandes G.C., Achatz M.I.W.S. (2014). Prevalence of the TP53 p.R337H Mutation in Breast Cancer Patients in Brazil. PLoS ONE.

[128] Seidinger A.L., Mastellaro M.J., Paschoal Fortes F., Godoy Assumpção J., Aparecida Cardinalli I., Aparecida Ganazza M., Correa Ribeiro R., Brandalise S.R., Dos Santos Aguiar S., Yunes J.A. (2011). Association of the highly prevalent TP53 R337H mutation with pediatric choroid plexus carcinoma and osteosarcoma in southeast Brazil. Cancer.

[129] Herrmann L.J., Heinze B., Fassnacht M., Willenberg H.S., Quinkler M., Reisch N., Zink M., Allolio B., Hahner S. (2012). TP53 germline mutations in adult patients with adrenocortical carcinoma. J. Clin. Endocrinol. Metab..

[130] Mirabello L., Zhu B., Koster R., Karlins E., Dean M., Yeager M., Gianferante M., Spector L.G., Morton L.M., Karyadi D. (2020). Frequency of Pathogenic Germline Variants in Cancer-Susceptibility Genes in Patients With Osteosarcoma. JAMA Oncol..

[131] Akouchekian M., Hemati S., Jafari D., Jalilian N., Dehghan Manshadi M. (2016). Does PTEN gene mutation play any role in Li-Fraumeni syndrome. Med. J. Islam. Repub. Iran.

[132] Saba K.H., Cornmark L., Kovac M., Magnusson L., Nilsson J., van den Bos H., Spierings D.C.J., Bidgoli M., Jonson T., Sumathi V.P. (2020). Oncogenes hijack a constitutively active *TP53promoter* in osteosarcoma. bioRxiv.

[133] Di Fiore R., Marcatti M., Drago-Ferrante R., D’Anneo A., Giuliano M., Carlisi D., De Blasio A., Querques F., Pastore L., Tesoriere G. (2014). Mutant p53 gain of function can be at the root of dedifferentiation of human osteosarcoma MG63 cells into 3AB-OS cancer stem cells. Bone.

[134] Zhou R., Xu A., Gingold J., Strong L.C., Zhao R., Lee D.F. (2017). Li-Fraumeni Syndrome Disease Model: A Platform to Develop Precision Cancer Therapy Targeting Oncogenic p53. Trends Pharm. Sci..

[135] Harvey M., McArthur M.J., Montgomery C.A., Butel J.S., Bradley A., Donehower L.A. (1993). Spontaneous and carcinogen-induced tumorigenesis in p53-deficient mice. Nat. Genet..

[136] Choi J., Curtis S.J., Roy D.M., Flesken-Nikitin A., Nikitin A.Y. (2010). Local mesenchymal stem/progenitor cells are a preferential target for initiation of adult soft tissue sarcomas associated with p53 and Rb deficiency. Am. J. Pathol..

[137] Lozano G. (2010). Mouse models of p53 functions. Cold Spring Harb. Perspect. Biol..

[138] Soussi T., Wiman K.G. (2015). TP53: An oncogene in disguise. Cell Death Differ..

[139] van Boxtel R., Kuiper R.V., Toonen P.W., van Heesch S., Hermsen R., de Bruin A., Cuppen E. (2011). Homozygous and Heterozygous p53 Knockout Rats Develop Metastasizing Sarcomas with High Frequency. Am. J. Pathol..

[140] Tong C., Li P., Wu N.L., Yan Y., Ying Q.L. (2010). Production of p53 gene knockout rats by homologous recombination in embryonic stem cells. Nature.

[141] Yan H.X., Wu H.P., Ashton C., Tong C., Ying Q.L. (2012). Rats deficient for p53 are susceptible to spontaneous and carcinogen-induced tumorigenesis. Carcinogenesis.

[142] Hansen S.A., Hart M.L., Busi S., Parker T., Goerndt A., Jones K., Amos-Landgraf J.M., Bryda E.C. (2016). Fischer-344 Tp53-knockout rats exhibit a high rate of bone and brain neoplasia with frequent metastasis. Dis. Model. Mech..

[143] Smyczyńska U., Strzemecki D., Czarnecka A.M., Fendler W., Fiedorowicz M., Wełniak-Kamińska M., Guzowska M., Synoradzki K., Cheda Ł., Rogulski Z. (2020). TP53-Deficient Angiosarcoma Expression Profiling in Rat Model. Cancers.

[144] Strzemecki D., Guzowska M., Grieb P. (2017). Survival rates of homozygotic Tp53 knockout rats as a tool for preclinical assessment of cancer prevention and treatment. Cell. Mol. Biol. Lett..

[145] McCoy A., Besch-Williford C.L., Franklin C.L., Weinstein E.J., Cui X. (2013). Creation and preliminary characterization of a Tp53 knockout rat. Dis. Model. Mech..

[146] Parant J.M., George S.A., Holden J.A., Yost H.J. (2010). Genetic modeling of Li-Fraumeni syndrome in zebrafish. Dis. Model. Mech..

[147] Ignatius M.S., Hayes M.N., Moore F.E., Tang Q., Garcia S.P., Blackburn P.R., Baxi K., Wang L., Jin A., Ramakrishnan A. (2018). tp53 deficiency causes a wide tumor spectrum and increases embryonal rhabdomyosarcoma metastasis in zebrafish. eLife.

[148] Regan D., Garcia K., Thamm D. (2018). Clinical, Pathological, and Ethical Considerations for the Conduct of Clinical Trials in Dogs with Naturally Occurring Cancer: A Comparative Approach to Accelerate Translational Drug Development. ILAR J..

[149] Rowell J.L., McCarthy D.O., Alvarez C.E. (2011). Dog models of naturally occurring cancer. Trends Mol. Med..

[150] Kirpensteijn J., Kik M., Teske E., Rutteman G.R. (2008). TP53 gene mutations in canine osteosarcoma. Vet. Surg. VS.

[151] Mori M., Hitora T., Nakamura O., Yamagami Y., Horie R., Nishimura H., Yamamoto T. (2015). Hsp90 inhibitor induces autophagy and apoptosis in osteosarcoma cells. Int. J. Oncol..

[152] McCleese J.K., Bear M.D., Fossey S.L., Mihalek R.M., Foley K.P., Ying W., Barsoum J., London C.A. (2009). The novel HSP90 inhibitor STA-1474 exhibits biologic activity against osteosarcoma cell lines. Int. J. Cancer.

[153] Roberts R.D., Lizardo M.M., Reed D.R., Hingorani P., Glover J., Allen-Rhoades W., Fan T., Khanna C., Sweet-Cordero E.A., Cash T. (2019). Provocative questions in osteosarcoma basic and translational biology: A report from the Children’s Oncology Group. Cancer.

[154] Samsa W.E., Mamidi M.K., Bashur L.A., Elliott R., Miron A., Chen Y., Lee B., Greenfield E.M., Chan R., Danielpour D. (2020). The crucial p53-dependent oncogenic role of JAB1 in osteosarcoma in vivo. Oncogene.

[155] Cortez M.A., Ivan C., Valdecanas D., Wang X., Peltier H.J., Ye Y., Araujo L., Carbone D.P., Shilo K., Giri D.K. (2015). PDL1 Regulation by p53 via miR-34. J. Natl. Cancer Inst..

[156] Corney D.C., Flesken-Nikitin A., Godwin A.K., Wang W., Nikitin A.Y. (2007). MicroRNA-34b and MicroRNA-34c are targets of p53 and cooperate in control of cell proliferation and adhesion-independent growth. Cancer Res..

[157] Chang T.C., Wentzel E.A., Kent O.A., Ramachandran K., Mullendore M., Lee K.H., Feldmann G., Yamakuchi M., Ferlito M., Lowenstein C.J. (2007). Transactivation of miR-34a by p53 broadly influences gene expression and promotes apoptosis. Mol. Cell.

[158] Slabáková E., Culig Z., Remšík J., Souček K. (2017). Alternative mechanisms of miR-34a regulation in cancer. Cell Death Dis..

[159] Wang Z., Zheng C., Jiang K., He J., Cao X., Wu S. (2017). MicroRNA-503 suppresses cell proliferation and invasion in osteosarcoma via targeting insulin-like growth factor 1 receptor. Exp. Ther. Med..

[160] Wen J., Zhao Y.-k., Liu Y., Zhao J.-f. (2017). MicroRNA-34a inhibits tumor invasion and metastasis in osteosarcoma partly by effecting C-IAP2 and Bcl-2. Tumor. Biol..

[161] Zhang Y., Pan Y., Xie C., Zhang Y. (2018). miR-34a exerts as a key regulator in the dedifferentiation of osteosarcoma via PAI-1-Sox2 axis. Cell Death Dis..

[162] Liang X., Xu C., Wang W., Li X. (2019). The DNMT1/miR-34a Axis Is Involved in the Stemness of Human Osteosarcoma Cells and Derived Stem-Like Cells. Stem Cells Int..

[163] Li Q.-C., Xu H., Wang X., Wang T., Wu J. (2018). miR-34a increases cisplatin sensitivity of osteosarcoma cells in vitro through up-regulation of c-Myc and Bim signal. Cancer Biomark..

[164] Beg M.S., Brenner A.J., Sachdev J., Borad M., Kang Y.-K., Stoudemire J., Smith S., Bader A.G., Kim S., Hong D.S. (2017). Phase I study of MRX34, a liposomal miR-34a mimic, administered twice weekly in patients with advanced solid tumors. Invest. New Drugs.

[165] Ganjavi H., Gee M., Narendran A., Parkinson N., Krishnamoorthy M., Freedman M.H., Malkin D. (2006). Adenovirus-mediated p53 gene therapy in osteosarcoma cell lines: Sensitization to cisplatin and doxorubicin. Cancer Gene Ther..

[166] Zhou X., Hao Q., Lu H. (2019). Mutant p53 in cancer therapy-the barrier or the path. J. Mol. Cell Biol..

[167] Duffy M.J., Synnott N.C., McGowan P.M., Crown J., O’Connor D., Gallagher W.M. (2014). p53 as a target for the treatment of cancer. Cancer Treat. Rev..

[168] Duffy M.J., Synnott N.C., Crown J. (2017). Mutant p53 as a target for cancer treatment. Eur. J. Cancer.

[169] Xia Y., Li X., Sun W. (2020). Applications of Recombinant Adenovirus-p53 Gene Therapy for Cancers in the Clinic in China. Curr. Gene Ther..

[170] Witlox M.A., Lamfers M.L., Wuisman P.I., Curiel D.T., Siegal G.P. (2007). Evolving gene therapy approaches for osteosarcoma using viral vectors: Review. Bone.

[171] Nakase M., Inui M., Okumura K., Kamei T., Nakamura S., Tagawa T. (2005). p53 gene therapy of human osteosarcoma using a transferrin-modified cationic liposome. Mol. Cancer Ther..

[172] Vassilev L.T., Vu B.T., Graves B., Carvajal D., Podlaski F., Filipovic Z., Kong N., Kammlott U., Lukacs C., Klein C. (2004). In vivo activation of the p53 pathway by small-molecule antagonists of MDM2. Science.

[173] Wang B., Fang L., Zhao H., Xiang T., Wang D. (2012). MDM2 inhibitor Nutlin-3a suppresses proliferation and promotes apoptosis in osteosarcoma cells. Acta Biochim. Biophys. Sin..

[174] Ray-Coquard I., Blay J.Y., Italiano A., Le Cesne A., Penel N., Zhi J., Heil F., Rueger R., Graves B., Ding M. (2012). Effect of the MDM2 antagonist RG7112 on the P53 pathway in patients with MDM2-amplified, well-differentiated or dedifferentiated liposarcoma: An exploratory proof-of-mechanism study. Lancet Oncol..

[175] Pishas K.I., Neuhaus S.J., Clayer M.T., Schreiber A.W., Lawrence D.M., Perugini M., Whitfield R.J., Farshid G., Manavis J., Chryssidis S. (2014). Nutlin-3a efficacy in sarcoma predicted by transcriptomic and epigenetic profiling. Cancer Res..

[176] Shangary S., Qin D., McEachern D., Liu M., Miller R.S., Qiu S., Nikolovska-Coleska Z., Ding K., Wang G., Chen J. (2008). Temporal activation of p53 by a specific MDM2 inhibitor is selectively toxic to tumors and leads to complete tumor growth inhibition. Proc. Natl. Acad. Sci. USA.

[177] Bill K.L., Garnett J., Meaux I., Ma X., Creighton C.J., Bolshakov S., Barriere C., Debussche L., Lazar A.J., Prudner B.C. (2016). SAR405838: A Novel and Potent Inhibitor of the MDM2:p53 Axis for the Treatment of Dedifferentiated Liposarcoma. Clin. Cancer Res..

[178] de Jonge M., de Weger V.A., Dickson M.A., Langenberg M., Le Cesne A., Wagner A.J., Hsu K., Zheng W., Mace S., Tuffal G. (2017). A phase I study of SAR405838, a novel human double minute 2 (HDM2) antagonist, in patients with solid tumours. Eur. J. Cancer.

[179] Tovar C., Graves B., Packman K., Filipovic Z., Higgins B., Xia M., Tardell C., Garrido R., Lee E., Kolinsky K. (2013). MDM2 small-molecule antagonist RG7112 activates p53 signaling and regresses human tumors in preclinical cancer models. Cancer Res..

[180] Zhang X., Wen X., Chen G., Zeng S., Men L., Wang H., He S., Ma Y., Pan Q., Zhang Y. (2020). Phase I study results of APG-115, a MDM2-p53 antagonist in Chinese patients with advanced liposarcoma and other solid tumors. J. Clin. Oncol..

[181] Konopleva M., Martinelli G., Daver N., Papayannidis C., Wei A., Higgins B., Ott M., Mascarenhas J., Andreeff M. (2020). MDM2 inhibition: An important step forward in cancer therapy. Leukemia.

[182] Parrales A., Iwakuma T. (2015). Targeting Oncogenic Mutant p53 for Cancer Therapy. Front. Oncol..

[183] Bykov V.J., Issaeva N., Shilov A., Hultcrantz M., Pugacheva E., Chumakov P., Bergman J., Wiman K.G., Selivanova G. (2002). Restoration of the tumor suppressor function to mutant p53 by a low-molecular-weight compound. Nat. Med..

[184] Wassman C.D., Baronio R., Demir O., Wallentine B.D., Chen C.K., Hall L.V., Salehi F., Lin D.W., Chung B.P., Hatfield G.W. (2013). Computational identification of a transiently open L1/S3 pocket for reactivation of mutant p53. Nat. Commun..

[185] Bykov V.J., Zache N., Stridh H., Westman J., Bergman J., Selivanova G., Wiman K.G. (2005). PRIMA-1(MET) synergizes with cisplatin to induce tumor cell apoptosis. Oncogene.

[186] Zache N., Lambert J.M.R., Rokaeus N., Shen J., Hainaut P., Bergman J., Wiman K.G., Bykov V.J.N. (2017). Mutant p53 targeting by the low molecular weight compound STIMA-1. Mol. Oncol..

[187] Kandoth C., McLellan M.D., Vandin F., Ye K., Niu B., Lu C., Xie M., Zhang Q., McMichael J.F., Wyczalkowski M.A. (2013). Mutational landscape and significance across 12 major cancer types. Nature.

[188] Liu X., Wilcken R., Joerger A.C., Chuckowree I.S., Amin J., Spencer J., Fersht A.R. (2013). Small molecule induced reactivation of mutant p53 in cancer cells. Nucleic Acids Res..

[189] Weinmann L., Wischhusen J., Demma M.J., Naumann U., Roth P., Dasmahapatra B., Weller M. (2008). A novel p53 rescue compound induces p53-dependent growth arrest and sensitises glioma cells to Apo2L/TRAIL-induced apoptosis. Cell Death Differ.

[190] Demma M., Maxwell E., Ramos R., Liang L., Li C., Hesk D., Rossman R., Mallams A., Doll R., Liu M. (2010). SCH529074, a small molecule activator of mutant p53, which binds p53 DNA binding domain (DBD), restores growth-suppressive function to mutant p53 and interrupts HDM2-mediated ubiquitination of wild type p53. J. Biol. Chem..

[191] Iyer S.V., Parrales A., Begani P., Narkar A., Adhikari A.S., Martinez L.A., Iwakuma T. (2016). Allele-specific silencing of mutant p53 attenuates dominant-negative and gain-of-function activities. Oncotarget.

[192] Parrales A., Ranjan A., Iyer S.V., Padhye S., Weir S.J., Roy A., Iwakuma T. (2016). DNAJA1 controls the fate of misfolded mutant p53 through the mevalonate pathway. Nat. Cell Biol..

[193] Wang C., Chen J. (2003). Phosphorylation and hsp90 binding mediate heat shock stabilization of p53. J. Biol. Chem..

[194] Li D., Marchenko N.D., Schulz R., Fischer V., Velasco-Hernandez T., Talos F., Moll U.M. (2011). Functional inactivation of endogenous MDM2 and CHIP by HSP90 causes aberrant stabilization of mutant p53 in human cancer cells. Mol. Cancer Res..

[195] Bekki H., Kohashi K., Maekawa A., Yamada Y., Yamamoto H., Harimaya K., Hakozaki M., Nabeshima K., Iwamoto Y., Oda Y. (2015). Elevated expression of HSP90 and the antitumor effect of an HSP90 inhibitor via inactivation of the Akt/mTOR pathway in undifferentiated pleomorphic sarcoma. BMC Cancer.

[196] Ory B., Baud’huin M., Verrecchia F., Royer B.B., Quillard T., Amiaud J., Battaglia S., Heymann D., Redini F., Lamoureux F. (2016). Blocking HSP90 Addiction Inhibits Tumor Cell Proliferation, Metastasis Development, and Synergistically Acts with Zoledronic Acid to Delay Osteosarcoma Progression. Clin. Cancer Res..

[197] Ozgur A. (2021). Investigation of anticancer activities of STA-9090 (ganetespib) as a second generation HSP90 inhibitor in Saos-2 osteosarcoma cells. J. Chemother..

[198] Shimamura T., Perera S.A., Foley K.P., Sang J., Rodig S.J., Inoue T., Chen L., Li D., Carretero J., Li Y.C. (2012). Ganetespib (STA-9090), a nongeldanamycin HSP90 inhibitor, has potent antitumor activity in in vitro and in vivo models of non-small cell lung cancer. Clin. Cancer Res..

[199] Li L., Wang L., You Q.D., Xu X.L. (2020). Heat Shock Protein 90 Inhibitors: An Update on Achievements, Challenges, and Future Directions. J. Med. Chem..

[200] Thoenen E., Curl A., Iwakuma T. (2019). TP53 in bone and soft tissue sarcomas. Pharmacology.

[201] Zhu J., Zou H., Yu W., Huang Y., Liu B., Li T., Liang C., Tao H. (2019). Checkpoint kinase inhibitor AZD7762 enhance cisplatin-induced apoptosis in osteosarcoma cells. Cancer Cell Int..

[202] Chou Y.S., Yen C.C., Chen W.M., Lin Y.C., Wen Y.S., Ke W.T., Wang J.Y., Liu C.Y., Yang M.H., Chen T.H. (2016). Cytotoxic mechanism of PLK1 inhibitor GSK461364 against osteosarcoma: Mitotic arrest, apoptosis, cellular senescence, and synergistic effect with paclitaxel. Int. J. Oncol..

[203] Mo H., He J., Yuan Z., Wu Z., Liu B., Lin X., Guan J. (2019). PLK1 contributes to autophagy by regulating MYC stabilization in osteosarcoma cells. OncoTargets Ther..

[204] Gutteridge R.E., Ndiaye M.A., Liu X., Ahmad N. (2016). Plk1 Inhibitors in Cancer Therapy: From Laboratory to Clinics. Mol. Cancer Ther..

[205] PosthumaDeBoer J., Wurdinger T., Graat H.C., van Beusechem V.W., Helder M.N., van Royen B.J., Kaspers G.J. (2011). WEE1 inhibition sensitizes osteosarcoma to radiotherapy. BMC Cancer.

[206] Kreahling J.M., Foroutan P., Reed D., Martinez G., Razabdouski T., Bui M.M., Raghavan M., Letson D., Gillies R.J., Altiok S. (2013). Wee1 inhibition by MK-1775 leads to tumor inhibition and enhances efficacy of gemcitabine in human sarcomas. PLoS ONE.

[207] Ghelli Luserna di Rora A., Cerchione C., Martinelli G., Simonetti G. (2020). A WEE1 family business: Regulation of mitosis, cancer progression, and therapeutic target. J. Hematol. Oncol..

[208] Zhang S., Zhou L., Hong B., van den Heuvel A.P., Prabhu V.V., Warfel N.A., Kline C.L., Dicker D.T., Kopelovich L., El-Deiry W.S. (2015). Small-Molecule NSC59984 Restores p53 Pathway Signaling and Antitumor Effects against Colorectal Cancer via p73 Activation and Degradation of Mutant p53. Cancer Res..

[209] Duan Z., Choy E., Harmon D., Yang C., Ryu K., Schwab J., Mankin H., Hornicek F.J. (2009). Insulin-like growth factor-I receptor tyrosine kinase inhibitor cyclolignan picropodophyllin inhibits proliferation and induces apoptosis in multidrug resistant osteosarcoma cell lines. Mol. Cancer Ther..

[210] Viereck V., Siggelkow H., Pannem R., Braulke T., Scharf J.G., Kübler B. (2007). Alteration of the insulin-like growth factor axis during in vitro differentiation of the human osteosarcoma cell line HOS 58. J. Cell. Biochem..

[211] Allison D.C., Carney S.C., Ahlmann E.R., Hendifar A., Chawla S., Fedenko A., Angeles C., Menendez L.R. (2012). A Meta-Analysis of Osteosarcoma Outcomes in the Modern Medical Era. Sarcoma.

[212] Marina N.M., Smeland S., Bielack S.S., Bernstein M., Jovic G., Krailo M.D., Hook J.M., Arndt C., van den Berg H., Brennan B. (2016). Comparison of MAPIE versus MAP in patients with a poor response to preoperative chemotherapy for newly diagnosed high-grade osteosarcoma (EURAMOS-1): An open-label, international, randomised controlled trial. Lancet Oncol..

[213] Gaspar N., Occean B.V., Pacquement H., Bompas E., Bouvier C., Brisse H.J., Castex M.P., Cheurfa N., Corradini N., Delaye J. (2018). Results of methotrexate-etoposide-ifosfamide based regimen (M-EI) in osteosarcoma patients included in the French OS2006/sarcome-09 study. Eur. J. Cancer.

[214] Brázda V., Fojta M. (2019). The Rich World of p53 DNA Binding Targets: The Role of DNA Structure. Int. J. Mol. Sci..

[215] Takahashi K., Yamanaka S. (2006). Induction of pluripotent stem cells from mouse embryonic and adult fibroblast cultures by defined factors. Cell.

[216] Frank A.K., Leu J.I.J., Zhou Y., Devarajan K., Nedelko T., Klein-Szanto A., Hollstein M., Murphy M.E. (2011). The Codon 72 Polymorphism of p53 Regulates Interaction with NF-κB and Transactivation of Genes Involved in Immunity and Inflammation. Mol. Cell Biol..

