# Supplementary material for: TP53 in Biology and Treatment of Osteosarcoma
Source: Cancers (Basel). 2021 Aug 25;13(17):4284. doi: 10.3390/cancers13174284 (PMC8428337; doi:10.3390/cancers13174284)
Supplement: Supplementary file 1 [file cancers-13-04284-s001.zip › cancers-1276149 Supplementary 9.8.pdf]

cancers

MDPI

Supplementary Materials: TP53 in Biology and Treatment of Osteosarcoma

Kamil Jozef Synoradzki, Ewa Bartnik, Anna M. Czarnecka, Michał Fiedorowicz, Wiktoria Firlej, Anna Brodziak, Agnieszka Stasinska, Piotr Rutkowski and Paweł Grieb

Table S1. List of missense mutations in the *TP53* gene founded in osteosarcoma derived patients samples. Description possess number of exon in which mutation is localize, codon number and literature reference. Abbreviation used are: Ref. for aminoacid which was changed and Alt. for altered aminoacid.

EXON	REF.	CODON	ALT.	LITERATURE
2	D	7	N	[79]
3	R	26	W	[79]
4	P	47	L / S	[80], [81]
4	P	72	R	[81]
4	Y	107	H	[81]
4	G	108	S	[81]
5	S	127	P	[82]
5	C	135	Y /F /R/W	[79], [83]
5	V	147	D	[79]
5	P	151	A	[81]
5	V	157	F	[83]
5	A	159	N	[84]
5	Y	163	C	[79]
5	V	172	N	[80]
5	V	173	M / G / L	[82,85], [79]
5	R	175	H	[79-81,85-87]
5	H	179	Q / Y	[88], [89]
5	R	181	P	[80]
5	S	185	N	[81]
6	H	193	Q / Y	[86,90], [83]
6	V	197	G	[80]
6	Y	205	C	[80,88,91]
6	S	215	I	[79]

6	Y	220	C	[80,83,85]
6	E	224	D	[85,89]
7	N	235	S	[81]
7	Y	236	C	[83]
7	M	237	I	[79,80,91]
7	C	238	F / G / Y	[79,92], [80]
7	S	241	Y	[86]
7	C	242	Y / R	[80-82], [81]
7	G	244	V	[86]
7	G	245	S / D	[80], [89]
7	M	246	T	[81]
7	R	248	Q / W /G	[80-83,88,89,91], [79,80,89], [83]
7	R	249	T	[82]
7	P	250	I / L / F	[80], [86,90], [87]
7	I	255	T / F	[92], [79]
7	T	256	S / P	[80,91], [82,83]
7	L	257	R	[83]
7	D	259	V	[86]
8	R	267	W	[82]
8	N	268	S	[87]
8	V	272	M	[85]
8	R	273	H / L / C	[80-82,85-90], [79,92], [85]
8	C	275	Y	[79,82,92]
8	A	276	P / G	[89], [79]
8	C	277	F	[82]
8	P	278	A	[81]
8	R	280	H	[80]
8	D	281	E / N / H / V	[79,83,90,92], [80,86,90], [80,85,86,90], [82,83]

8	R	282	Q / W	[89], [81,83]
8	E	285	K	[80,88]
8	E	286	K	[81]
8	R	290	C	[82]
8	E	298	K	[81]
10	R	337	H / C / L	[82,88], [79,80,83], [82]

Table S2. Overview of clinical trials retrieved from ClinicalTrials.gov and EU Clinical Trials Register. The search was performed with keywords: p53, gene therapy. Trials with status "withdrawn" were excluded. Databases accessed on 10/22/2020.

Trial No.	Condition/group of patients	Administered agent	Phase	Start (year)	Status	Enrollment	Locations
NCT02561546	Diabetes concurrent with Hepatocellular carcinoma (HCC).	p53 gene therapy, Transcatheter embolization (TAE)	2	2015	Unknown	40	China, Liaoning<br>first affiliated hospital in Dalian University<br>Dalian, Liaoning, China
NCT02509169	Advanced Hepatocellular Carcinoma (HCC)	TAE plus P53 gene therapy	2	2014	Unknown	60	China, Liaoning<br>first affiliated hospital in Dalian University<br>Dalian, Liaoning, China
NCT01574729	Non-small Cell Lung Cancer	Surgery combined with rAd-p53 gene therapy	2	2012	Unknown	120	Institute of Surgery Research, Daping Hospital, Third Military Medical University Chongqing, Chongqing, China
NCT02435186	Ovarian Epithelial Cancer, Fallopian Tube Cancer, Primary Peritoneal Cancer	p53 gene, Cisplatin, Paclitaxel	2	2015	Unknown	100	Xijing Hospital in China Medical University Shenyang Liaoning, China,
NCT00902083	Advanced Oral and Maxillofacial Malignant Tumors	-p53 gene with surgery<br>-p53 gene with standard chemotherapy<br>-p53 gene therapy	4	2009	Unknown	600	West China Hospital, Sichuan University<br>Chengdu, Sichuan, China
NCT02429037	Advanced Head and Neck Cancer	- radiation and Cisplatin<br>-rAd-p53 and radiation and Cisplatin	2	2015	Unknown	60	Jiangsu cancer hospital<br>Nanjing, Jiangsu, China

NCT00902122	Advanced Malignant Thyroid Tumors	-rAd-p53<br>- rAd-p53 with surgery<br>-rAd-p53 with radiatioactive iodine	4	2009	Unknown	600	West China Hospital, Sichuan University Chengdu, Sichuan, China
NCT02429726	Malignant Pleural Effusion	-rAdp53<br>-Cisplating<br>- rAdp53 plus cisplatin	2	2015	Unknown	90	The First Affiliated Hospital of Xi'an Jiao Tong University<br>Xian, Shanxi, China
NCT00064103	-Lip and Oral Cavity Cancer<br>-Oropharyngeal Cancer Stage 0<br>-Lip and Oral Cavity Cancer Stage 0<br>-Oropharyngeal Cancer<br>-Tongue Cancer	Ad5CMV-p53 gene	2	2003	Completed	51	M D Anderson Cancer Center Houston, Texas, United States
NCT02418988	Advanced Adult Hepatocellular Carcinoma	-TACE plus rAd-p53 artery injection<br>- TACE	2	2014	Unknown	120	China, Shanxi<br>Xijing Hospital of the Fourth Military Medical University<br>Xi An, Shanxi, China

NCT00001827	Ovarian Neoplasm	-Aldesleukin<br>-incomplete Freund's adjuvant<br>-p53 peptide vaccine<br>-Sargramostim<br>-therapeutic autologous dendritic cells<br>Procedure: in vitro-treated peripheral blood stem cell transplantation	2	1999	Terminated	21	United States, Maryland<br>National Institutes of Health Clinical Center, Bethesda, Maryland, United States

NCT01191684	Recurrent Colon Cancer<br>Recurrent Gastric Cancer<br>Recurrent Pancreatic Cancer<br>Recurrent Rectal Cancer<br>Stage III Colon Cancer<br>Stage III Gastric Cancer<br>Stage III Pancreatic Cancer<br>Stage III Rectal Cancer<br>Stage IV Colon Cancer<br>Stage IV Gastric Cancer<br>Stage IV Pancreatic Cancer<br>Stage IV Rectal Cancer	modified vaccinia virus ankara vaccine expressing p53	1	2011	Completed	12	City of Hope Medical Center<br>Duarte, California, United States
NCT00003450	Ovarian Cancer<br>Peritoneal Cavity Cancer	Ad5CMV-p53 gene	1	1998	Completed	15-20	Simmons Cancer Center - Dallas<br>Dallas, Texas, United States

NCT00894153	head and neck malignant tumors in advanced stage	-rAd-p53 intra-tumor injection<br>- rAd-p53 intra-tumor injectionplus chemotherapy<br>- rAd-p53 intra-tumor injection plus radiotherapy<br>- rAd-p53 intra-tumor injection plus surgery	4	2009	Unknown	1200	Department of oral and maxillofacial surgery, Huaxi university of medical science<br>Chendu, Shichuan, China
NCT00004041	Brain and Central Nervous System Tumors	intratumoral stereotactic injection of adenovirus p53 (Ad-p53)	1	1999	Completed	30	UCSF Cancer Center and Cancer Research Institute<br>San Francisco, California, United States
NCT00004038	Breast Cancer	Ad5CMV-p53 gene plus chemotherapy	1	1999	Completed	20	Fox Chase Cancer Center<br>Philadelphia, Pennsylvania, United States
NCT00003147	Liver Cancer	Ad5CMV-p53 gene	1	1998	Terminated	30	Albert Einstein Comprehensive Cancer Center<br>Bronx, New York, United States
NCT00410865	Mouth Cancer	INGN 201 (Ad-p53 gene)	1	2003	Terminated	4	UT MD Anderson Cancer Center<br>Houston, Texas, United States

NCT00003167	-Recurrent Bladder Cancer:<br>-Stage I<br>-Stage II<br>- Stage III<br>- Stage IV<br>-Transitional Cell Carcinoma of the Bladder	Ad5CMV-p53 gene	1	1998	Completed	24	M D Anderson Cancer Center<br>Houston, Texas, United States
NCT00003649	Lung Cancer	Ad5CMV-p53 gene	1	1998	Completed	15	Veterans Affairs Medical Center - Tennessee Valley Healthcare System - Nashville Campus<br>Nashville, Tennessee, United States<br>Vanderbilt-Ingram Cancer Center<br>Nashville, Tennessee, United States
NCT00003588	Ovarian Cancer	Ad5CMV-p53 gene (laparoscopic surgery)	1	1998	Completed	30	University of Texas - MD Anderson Cancer Center<br>Houston, Texas, United States
NCT03544723	Solid Tumor<br>Lymphoma	Ad-p53	2	2018	Recruiting	40	Robert H. Lurie Comprehensive Cancer Center | Northwestern University<br>Chicago, Illinois, United States

NCT00003257	Head and Neck Cancer	Ad5CMV-p53 gene	2	1998	Unknown	39	Sidney Kimmel Cancer Center<br>San Diego, California, United States,<br><br>University of Colorado Cancer Center<br>Denver, Colorado, United States<br><br>University of Connecticut School of Medicine<br>Farmington, Connecticut, United States<br><br>Clinical Sciences Building<br>Chicago, Illinois, United States<br><br>University of Iowa Hospitals and Clinics<br>Iowa City, Iowa, United States<br><br>University of Kansas Medical Center<br>Kansas City, Kansas, United States<br><br>Tulane University School of Medicine<br>New Orleans, Louisiana, United States<br><br>Marlene & Stewart Greenebaum Cancer Center, University of Maryland<br>Baltimore, Maryland, United States<br><br>Albert Einstein Comprehensive Cancer Center<br>Bronx, New York, United States<br><br>Simmons Cancer Center - Dallas<br>Dallas, Texas, United States

NCT00017173	Head and Neck Cancer	Ad5CMV-p53 gene, cisplatin, conventional surgery, radiation therapy	2	2003	Terminated	13	Kansas Masonic Cancer Research Institute at the University of Kansas Medical Center<br>Kansas City, Kansas, United States<br><br>Markey Cancer Center at University of Kentucky Chandler Medical Center<br>Lexington, Kentucky, United States<br><br>Barbara Ann Karmanos Cancer Institute<br>Detroit, Michigan, United States
NCT02340156	Recurrent Glioblastoma	SGT53, Temozolomide	2	2014	Terminated	1	MD Anderson Cancer Center<br>Houston, Texas, United States<br><br>China Medical University Hospital<br>Taichung, Taiwan
NCT02340117	Metastatic Pancreatic Cancer	SGT-53,nab-paclitaxel, Gemcitabine	2	2015	Recruiting	28	Mary Crowley Cancer Research Center<br>Dallas, Texas, United States
NCT00049218	Lung Cancer	Autologous dendritic cell-adenovirus p53 vaccine, Carboplatin, Etoposide	2	2003	Completed	56	H. Lee Moffitt Cancer Center and Research Institute<br>Tampa, Florida, United States
NCT00617409	Small Cell Lung Cancer	Paclitaxel, Ad.p53-DC vaccines, All -trans Retinoic Acid (ATRA)	2	2007	Completed	69	H. Lee Moffitt Cancer Center & Research Institute<br>Tampa, Florida, United States

NCT00004225	Lung Cancer	Ad5CMV-p53 gene plus radiation therapy	1	2000	Completed	10	Vanderbilt-Ingram Cancer Center at Vanderbilt Medical Center Nashville, Tennessee, United States
NCT00044993	Breast Cancer	-Ad5CMV-p53 gene plus docetaxel plus doxorubicin hydrochloride -conventional surgery (neoadjuvant therapy)	2	2002	Completed	60	University of Texas - MD Anderson Cancer Center Houston, Texas, United States
NCT00393029	Metastatic Cancer That Overexpresses p53 Using Lymphodepleting Conditioning Followed by Infusion of Anti-p53 T Cell Receptor (TCR)-Gene Engineered Lymphocytes	-Anti-protein 53 or tumor protein 53 (p53) T-cell receptor transduced peripheral blood lymphocytes -Aldesleukin -Filgrastim -Cyclophosphamide -fludarabine phosphate	2	2006	Completed	12	National Institutes of HealthBethesda, Maryland, United States
NCT00004080	Brain and Central Nervous System Tumors (recurrent and progressive)	-recombinant adenovirus-p53 SCH-58500 conventional surgery	1	1999	Completed	21-42	Emory University, Atlanta, United States

NCT00002960	Fallopian Tube Cancer<br>Metastatic Cancer<br>Ovarian Cancer<br>Primary Peritoneal Cavity Cancer	recombinant adenovirus-p53 SCH-58500	1	1999	Completed	59	Merck Sharp & Dohme Corp., United Sates, Germany
NCT00041613	Carcinoma, Squamous Cell	INGN 201	3		Unknown		University of Colorado Cancer Center<br>Aurora, Colorado, United States
NCT00041626	Carcinoma, Squamous Cell	INGN 201	3		Unknown		University of Arkansas, Little Rock, Arkansas, United States
NCT00776295	Small Cell Lung Cancer	Combined adenovirus vectored **p53** transfected dendritic cell vaccine and ex vivo expanded T-lymphocytes	2	2007	Terminated	2	H. Lee Moffitt Cancer Center & Research Institute, Tampa, Florida, USA
NCT02275039	Recurrent Ovarian Epithelial Cancer<br>Recurrent Fallopian Tube Carcinoma<br>Recurrent Primary Peritoneal Carcinoma	-modified vaccinia virus ankara vaccine expressing **p53-**gemcitabine hydrochloride	1	2015	Completed	12	City of Hope Medical Center<br>Duarte, California, United States
NCT00844506	Ovarian Cancer	**P53-**SLP vaccine<br>Cyclophosphamide	2	2008	Completed	19	University Medical Centre Groningen<br>Groningen, Netherlands

NCT03406715	Small Cell Lung Cancer Lung Cancer Relapsed Small Cell Lung Cancer	Nivolumab<br>Ipilimumab<br>Dendritic Cell based **p53** Vaccine	2	2018	Active, not recruiting	14	H. Lee Moffitt Cancer Center and Research Institute, Tampa, Florida, United States
NCT04116541	Malignant Solid Tumor	HDM201 Ribociclib<br>Cabozantinib<br>Alectinib	2	2020	Recruiting	100	Centre Léon Bérard<br>Lyon, France<br><br>Institut Paoli Calmettes<br>Marseille, France<br>Centre Antoine LACASSAGNENice, France<br>Institute Curie<br>Paris, France<br>Institute Claudius Regaud<br>Toulouse, France
NCT01029873	Metastatic Melanoma	Cisplatin<br>ALT-801	1<br>2	2010	Completed	25	The Angeles Clinic and Research Institute<br>Los Angeles, California, United States<br>MD Anderson Cancer Center Orlando Orlando, Florida, United States<br>Emory University<br>Atlanta, Georgia, United States,<br>United States, Illinois<br><br>Northwestern University<br>Chicago, Illinois, United States<br>United States, Iowa<br>University of Iowa Hospitals and Clinics United States, Iowa<br>Iowa City, Iowa, United States, University of Iowa Hospitals and Clinics<br>United States, North CarolinaIowa City, Iowa, United States

	Carolinas Medical Center-Brumenthal Cancer Center United States, North Carolina Carolinas Medical Center-Brumenthal Cancer Center, Charlotte, North Carolina, United States,
	St. Luke's Hospital and Health Network, Pennsylvania, United States St. Luke's Hospital and Health Network, Bethlehem, Pennsylvania, United States,
	United States, Washington University of Washington, Seattle Cancer Care Center United States, Washington Seattle, Washington, United States, University of Washington, Seattle Cancer Care Center United States, ColoradoSeattle, Washington, United States University of Colorado, Anschutz Cancer Pavil-lionUnited States, Colorado
	United States, Washington United States, WashingtonUniversity of Washington, Seattle Cancer Care Center

NCT00496860	Progressive Metastatic Malignancies	ALT-801	1	2007	Completed	26	University of Colorado, Anschutz Cancer, Aurora, Colorado, United States States<br><br>University of Washington, Seattle Cancer Care Center Seattle, Washington, United States,United States, ColoradoUniversity of Colorado, Anschutz Cancer Pavillion<br><br>MD Anderson Cancer Center Orlando, Florida, United States<br>H. Lee Moffitt Cancer Center & Research Institute United States;,University of Colorado, Anschutz Cancer PavillionAurora, Colorado, United States Tampa, Florida, United States, H. Lee Moffitt Cancer Center & Research InstituteAurora, Colorado, United States,United States,Florida<br>Seattle Cancer Care Center United States,

NCT02432963	Adult Solid Neoplasm<br>Bladder Carcinoma<br>Colon Carcinoma<br>Estrogen Receptor Negative<br>Head and Neck Squamous Cell Carcinoma<br>Hepatocellular Carcinoma<br>HER2/Neu Negative Melanoma<br>Non-Small Cell Lung Carcinoma<br>Pancreatic Carcinoma<br>Progesterone Receptor Negative<br>Rectal Carcinoma<br>Renal Cell Carcinoma<br>Soft Tissue Sarcoma<br>Triple-Negative Breast Carcinoma<br>**TP53 Gene** Mutation<br>Unresectable Solid Neoplasm	Modified Vaccinia Virus Ankara Vaccine Expressing **p53**Pembrolizumab	1	2016	Active,not recruiting	19	City of Hope Medical Center Duarte, California, United States

NCT01639885	Recurrent Ovarian Cancer	Interferon Alfa-2b<br>**p53** SLP	1<br>2	2011	Completed	15	Leiden University Medical Center<br>Leiden, Netherlands
NCT01670994	Relapsed or Refractory Multiple Myeloma	ALT-801	1<br>2	2012	Terminated	6	University of Iowa Hospitals,<br>Iowa City, Iowa, United States
NCT01625260	Non-muscle Invasive Bladder Cancer	ALT-801<br>Gemcitabine	1<br>2	2012	Unknown	52	University of Alabama Comprehensive Cancer Center<br>Birmingham, Alabama, United States<br>University of California Davis<br>Sacramento, California, United States<br><br>UF Health Center at Orlando Health<br>Orlando, Florida, United States<br>University of Oklahoma Health Science Center<br>Oklahoma City, Oklahoma, United States<br>UPMC Cancer Center<br>Pittsburgh, Pennsylvania, United States,
NCT03725436	Advanced Malignant Solid Neoplasm<br>Anatomic Stage III Breast Cancer AJCC v8<br>Anatomic Stage IIIA Breast Cancer AJCC v8<br>Anatomic Stage IIIB Breast Cancer AJCC v8	MDM2/MDMX Inhibitor<br>ALRN-6924<br>Paclitaxel	1	2019	Recruiting	45	M D Anderson Cancer Center<br>Houston, Texas, United States,

Anatomic Stage IIIC Breast Cancer AJCC v8 Estrogen Receptor PositiveHER2/Neu NegativeMetastatic Malignant Solid Neoplasm Prognostic Stage III Breast Cancer AJCC v8 Prognostic Stage IIIA Breast Cancer AJCC v8 Prognostic Stage IIIB Breast Cancer AJCC v8 Prognostic Stage IIIC Breast Cancer AJCC v8 Recurrent Breast Carcinoma **TP53 wt Allele** Unresectable Malignant Solid Neoplasm

NCT01326871	Transitional Cell Carcinoma of Bladder<br>Urethra Cancer<br>Ureter Cancer<br>**Malignant Tumor of Renal Pelvis**	Cisplatin Gemcitabine<br>ALT-801	1<br>2	2011	Unknown	90	The University of Arizona Cancer Center<br>Tucson, Arizona, United States,<br><br>UF Health Center at Orlando Health<br>Orlando, Florida, United States,<br>Martin Health System<br>Stuart, Florida, United States,<br>H. Lee Moffitt Cancer Center and Research Institute<br>Tampa, Florida, United States,<br><br>Emory University<br>Atlanta, Georgia, United States,<br><br>Robert Lurie Comprehensive Cancer Center of Northwestern University<br>Chicago, Illinois, United States,<br><br>University of Iowa Hospitals and Clinics<br>Iowa City, Iowa, United States,<br><br>University of Kansas Cancer Center<br>Fairway, Kansas, United States,<br><br>Karmanos Cancer Center<br>Detroit, Michigan, United States,<br><br>University of Minnesota<br>Minneapolis, Minnesota, United States,<br><br>Washington University<br>St. Louis, Missouri, United States,<br><br>Levine Cancer Institute<br>Charlotte, North Carolina, United States,

							University of Oklahoma Health Science Center<br>Oklahoma City, Oklahoma, United States,<br><br>St. Luke's Hospital and Health Network<br>Easton, Pennsylvania, United States,<br><br>Thomas Jefferson University Hospital<br>Philadelphia, Pennsylvania, United States,<br><br>UPMC Cancer Center<br>Pittsburgh, Pennsylvania, United States,
NCT03113487	Recurrent Platinum-Resistant Fallopian Tube Carcinoma<br>Recurrent Platinum-Resistant Ovarian Carcinoma<br>Recurrent Platinum-Resistant Primary Peritoneal Carcinoma	Modified Vaccinia Virus Ankara Vaccine Expressing **p53**Pembrolizumab	2	2018	Recruiting	28	United States, California<br>City of Hope Medical Center<br>Duarte, California, United States,
